# Four new *Gammarus* species from Tibetan Plateau with a key to Tibetan freshwater gammarids (Crustacea, Amphipoda, Gammaridae)

**DOI:** 10.3897/zookeys.747.21999

**Published:** 2018-03-28

**Authors:** Zhonge Hou, Shuqiang Li

**Affiliations:** 1 Key Laboratory of Zoological Systematics and Evolution, Institute of Zoology, Chinese Academy of Sciences, Beijing 100101, China; 2 Southeast Asia Biodiversity Research Institute, Chinese Academy of Sciences, Yezin, Nay Pyi Taw 05282, Myanmar

**Keywords:** Amphipoda, diversification, freshwater, taxonomy

## Abstract

Four new species of the genus *Gammarus* are described and illustrated from Tibetan Plateau. *Gammarus altus* **sp. n.** and *G. limosus* **sp. n.** are characterized by pereopods III–IV with a few short setae and uropod III with marginal spines accompanied by short setae. *Gammarus kangdingensis* **sp. n.** and *G. gonggaensis* **sp. n.** are characterized by pereopods III–IV with long straight setae on posterior margins and inner ramus of uropod III 0.4 times as long as outer ramus. Detailed morphological comparisons with related species are discussed. A key to 15 *Gammarus* species from the Tibetan Plateau and a map of their distributions are provided.

## Introduction

The Tibetan Plateau is the highest and largest plateau in the world, and arguably the most prominent topological feature on Earth (Royden et al. 2008, Favre et al. 2015). The Tibetan Plateau is typically inhabited by highly specialized biota, which is adapted to the extreme environmental conditions, such as wild yaks and Tibetan gazelles (Hou and Li 2004). However, the freshwater diversity of the Tibetan Plateau remains poorly understood, except fishes (He and Chen 2006, Qi et al. 2015) and mollusks (Clewing et al. 2015, 2016a). In order to better understand the biodiversity of the Tibetan Plateau, six expeditions were organized by the authors from 2001 to 2017. Following a detailed examination of the collections, crustaceans of the genus *Gammarus* Fabricius, 1775 were found widely distributed in alpine lakes and river systems. All species are cold-water adapted.

**Figure 1.**
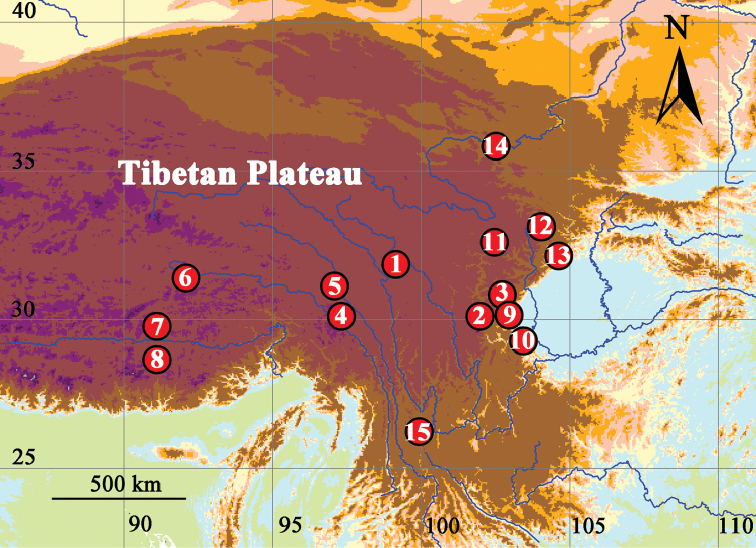
Distribution map of *Gammarus* species from Tibetan Plateau. Type localites are shown for species **1–14**. **1** *Gammarus altus* sp. n. **2** *G. kangdingensis* sp. n. **3** *G. gonggaensis* sp. n. **4** *G. limosus* sp. n. **5** *G. sinuolatus* Hou & Li, 2004 **6** *G. frigidus* Hou & Li, 2004 **7** *G. lasaensis* Barnard & Dai, 1988 **8** *G. jaspidus* Hou & Li, 2004 **9** *G. abstrusus* Hou, Platvoet & Li, 2006 **10** *G. emeiensis* Hou, Li & Koenemann, 2002 **11** *G. hongyuanensis* Barnard & Dai, 1988 **12** *G. sichuanensis* Hou, Li & Zheng, 2002 **13** *G. praecipuus* Li, Hou & An, 2013 **14** *G. glaber* Hou, 2017 **15** *Gammarus lacustris* Sars, 1863 based on material used in Barnard and Dai (1988).

The Holarctic amphipod genus *Gammarus* contains more than 200 described species (Väinölä et al. 2008). It has been suggested that *Gammarus* originated from the ancient Tethys, and then diversified in the Eurasia driven by the Tibetan uplifting (Hou et al. 2007, 2011, Hou and Li 2017). Eleven species were described from the plateau before the current study: ten are endemic species, including *G. sinuolatus* Hou & Li, 2004, *G. frigidus* Hou & Li, 2004, *G. lasaensis* Barnard & Dai, 1988, *G. jaspidus* Hou & Li, 2004, *G. abstrusus* Hou, Platvoet & Li, 2006, *G. emeiensis* Hou, Li & Koenemann, 2002, *G. hongyuanensis* Barnard & Dai, 1988, *G. sichuanensis* Hou, Li & Zheng, 2002, *G. praecipuus* Li, Hou & An, 2013, and *G. glaber* Hou, 2017 (Zhao et al. 2017); while *G. lacustris* Sars, 1863 is widely distributed in alpine lakes (Karaman and Pinkster 1977, Clewing et al. 2016b). In the current paper, four new species are described and illustrated: *Gammarus altus* sp. n., *G. kangdingensis* sp. n., *G. gonggaensis* sp. n., and *G. limosus* sp. n. The distributions of 15 *Gammarus* species from Tibetan Plateau are presented in Figure 1, where only type localities are used for all species except *G. lacustris* Sars, 1863 based on material of Barnard and Dai (1988). A key to all these species is provided.

## Materials and methods

The specimens were collected along the bank of streams flowing from high mountains with a fine-meshed hand net. Samples were preserved in 95% ethanol in the field and deposited in a -20°C refrigerator for long-term preservation. The body length of each amphipod was recorded by holding the specimen straight and measuring the distance along the dorsal side of the body from the base of the first antenna to the base of the telson. All dissected appendages were mounted in glycerol on slides. Appendages were drawn using a Leica DM2500 compound microscope equipped with a drawing tube. The specimens are lodged in the Institute of Zoology, Chinese Academy of Sciences (**IZCAS**), Beijing.

## Taxonomy

### Family Gammaridae Leach, 1814

#### Genus Gammarus

Taxon classificationAnimaliaORDOFAMILIA

Fabricius, 1775

##### Type species.

*Gammarus pulex* (Linnaeus, 1758).

#### Gammarus altus sp. n.

Taxon classificationAnimaliaORDOFAMILIA

http://zoobank.org/B32F6C92-AEAB-4A62-BD6F-4916FCC73377

**Figure 2.**
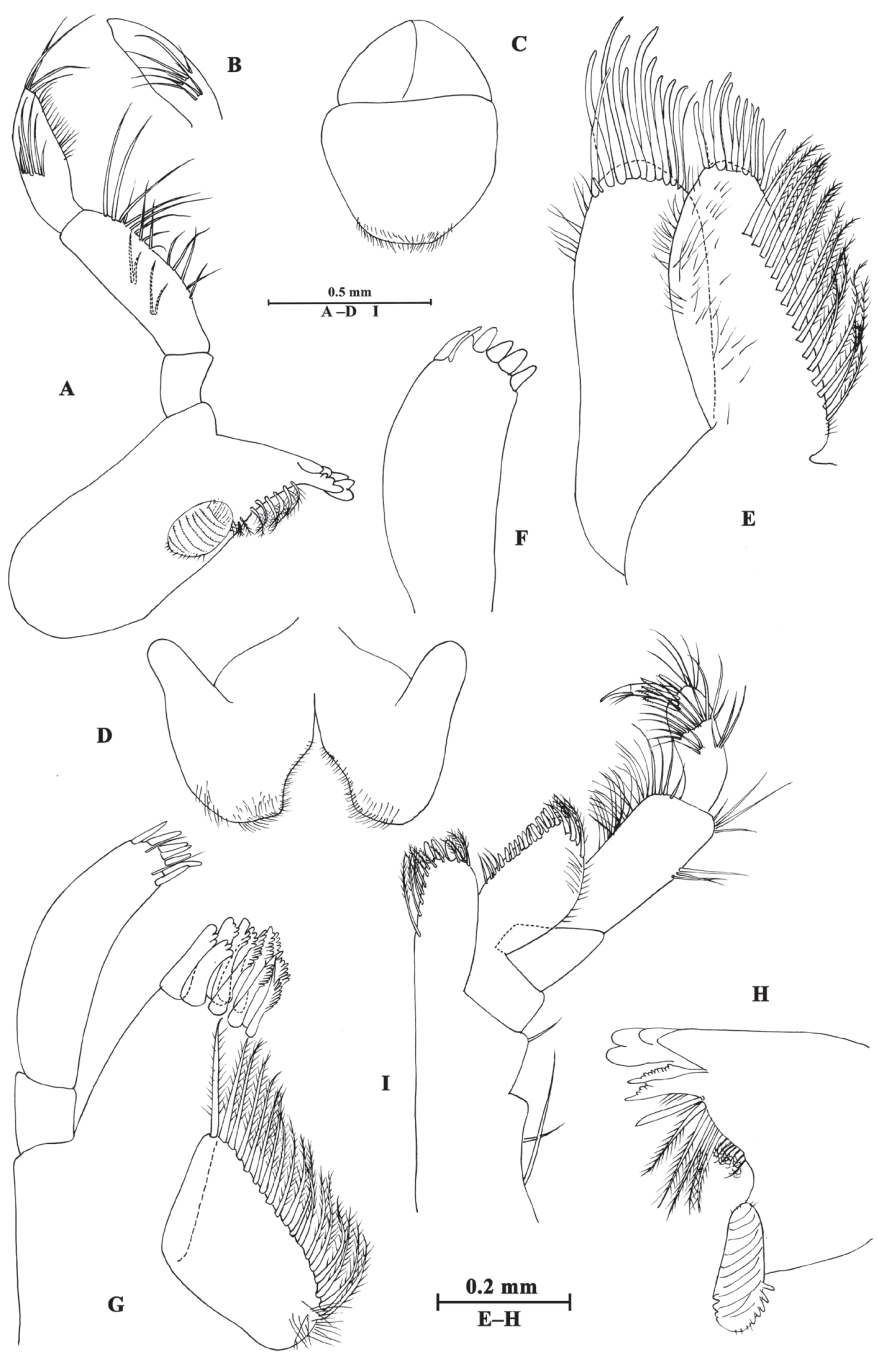
*Gammarus altus* sp. n., male holotype. **A** left mandible **B** inner face of article III of left palp **C** upper lip **D** lower lip **E** maxilla II **F** palp of right maxilla I **G** left maxilla I **H** incisor of right palp **I** maxilliped.

**Figure 3.**
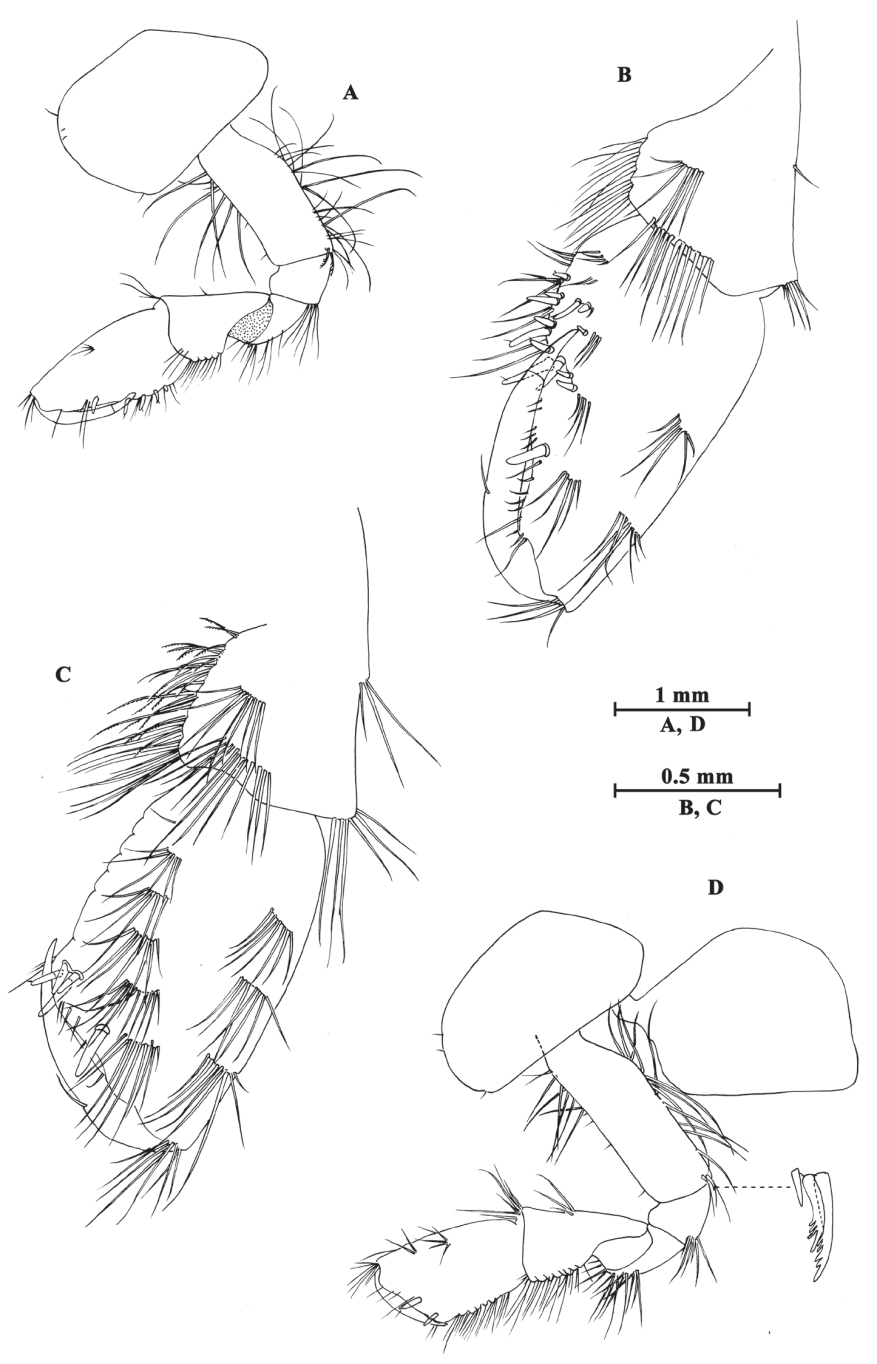
*Gammarus altus* sp. n., male holotype. **A** gnathopod I **B** propodus of gnathopod I (medial view) **C** propodus of gnathopod II (medial view) **D** gnathopod II.

**Figure 4.**
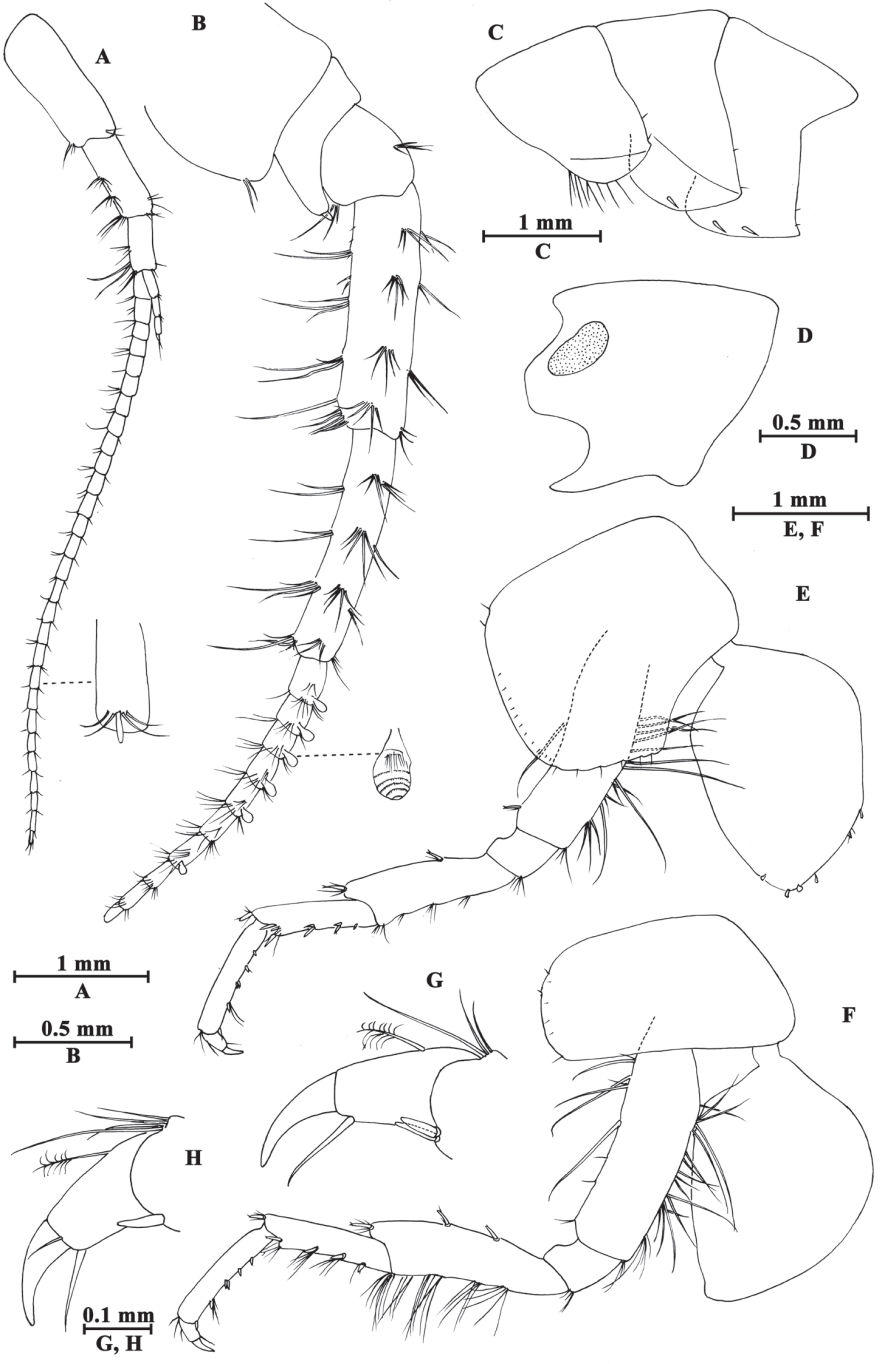
*Gammarus altus* sp. n., male holotype. **A** antenna I **B** antenna II **C** epimeral plates I–III **D** head **E** pereopod IV **F** pereopod III **G** dactylus of pereopod III **H** dactylus of pereopod IV.

**Figure 5.**
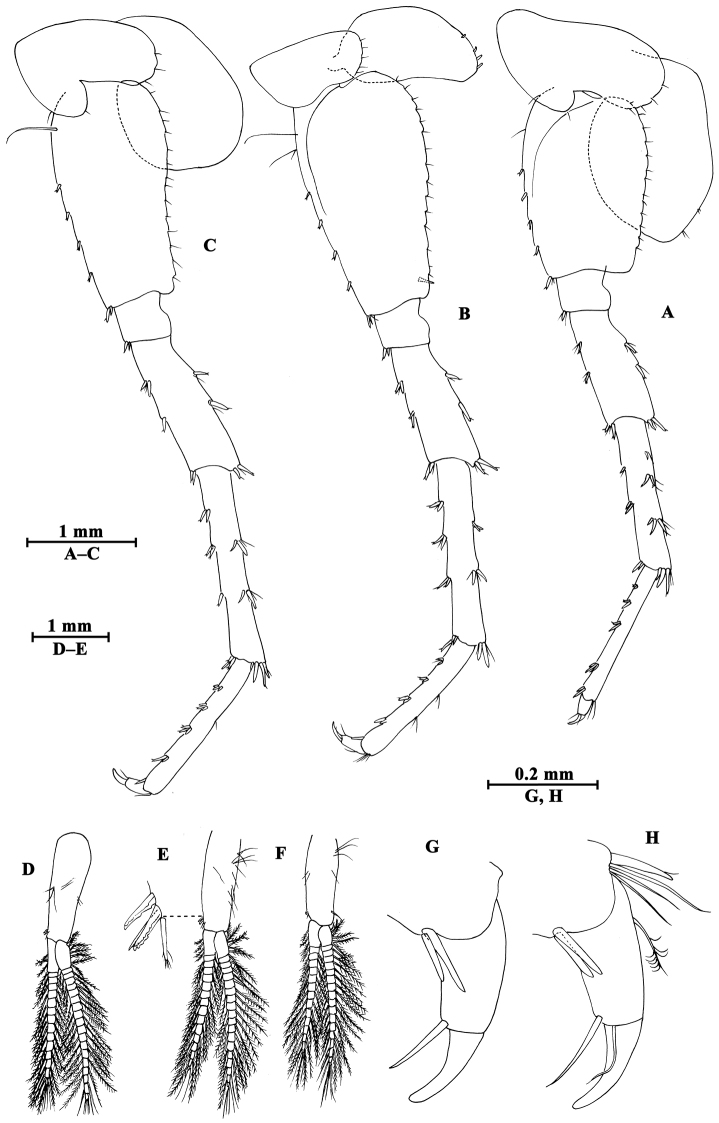
*Gammarus altus* sp. n., male holotype. **A** pereopod V **B** pereopod VII **C** pereopod VI **D** pleopod I **E** pleopod II **F** pleopod III **G** dactylus of pereopod VI **H** dactylus of pereopod VII.

**Figure 6.**
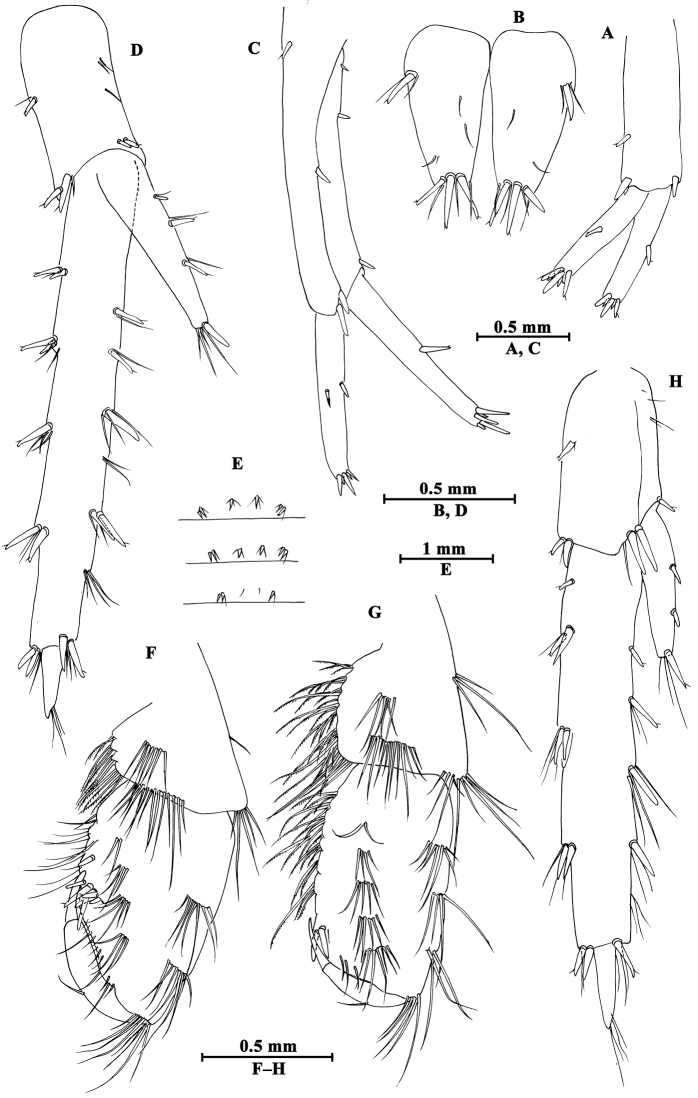
*Gammarus altus* sp. n., **A–E** male, holotype **F–H** female, paratype. **A** uropod II **B** telson **C** uropod I **D** uropod III **E** urosomites I–III (dorsal view) **F** propodus of gnathopod I (medial view) **G** propodus of gnathopod II (medial view) **H** uropod III.

Figs 2 , 3 , 4 , 5 , 6

##### Material examined.

Holotype: male (IZCAS-I-A0061-1), 11.6 mm, Maniganggo Town (31.9°N, 99.2°E), Dege County, Sichuan Province, altitude 4000 m, August 12, 2001, collected by Xianjin Peng. Paratype: female (IZCAS-I-A0061-2), 10.1 mm; paratypes: five males and five females (IZCAS-I-A0061-3), same data as holotype.

##### Etymology.

The specific name alludes to its typical biotope, living in high altitude; adjective.

##### Diagnosis.

Pereopods III and IV with a few setae on posterior margins; pereopods V–VII slender, bases elongated, carpus and propodus with spines on anterior margins but few setae; epimeral plates with blunt posterodistal corners; uropod III inner ramus length approx. one-third of outer ramus length, both rami with marginal spines but with few marginal setae.

##### Description of male holotype.

(IZCAS-I-A0061-1), 11.6 mm.

**Head.** (Fig. 4D): cephalic lateral lobe truncated, inferior antennal sinus deep, eyes ovate.

Antenna I (Fig. 4A): peduncle articles I–III in length ratio 1.0 : 0.7 : 0.4, with some setae on posterior margin; primary flagellum with 28 articles, most with aesthetascs, accessory flagellum with three articles.

Antenna II (Fig. 4B): gland cone reaching peduncle article III, article IV a little shorter than article V, bearing one to three clusters of setae on lateral and medial margins; flagellum with ten articles, proximal seven articles with calceoli.

Upper lip (Fig. 2C): convex, with minute setae.

Mandible (Fig. 2A, B, H): incisor of left mandible with five teeth; lacinia mobilis with four teeth; spine row with six plumose setae; second article of palp with 15 marginal setae and four medial setae, third article 83% length of second article, with six A-setae on outer face, two groups of B-setae on inner face, approx. 17 D-setae and four E-setae apically. Incisor of right mandible with four teeth; lacinia mobilis bifurcate, with small teeth.

Lower lip (Fig. 2D): inner lobes lacking, outer lobes covered with thin setae.

Maxilla I (Fig. 2F, G): asymmetrical, left inner plate with 18 plumose setae; outer plate with eleven serrated apical spines; second article of left palp with seven slender spines and two stiff setae; second article of right palp with five blunt spines and one stiff seta.

Maxilla II (Fig. 2E): inner plate with 16 plumose setae in an oblique row; inner and outer plates with long setae apically.

Maxilliped (Fig. 2I): inner plate with three apical spines and 12 plumose marginal setae; outer plate with a row of 14 blade spines on medial margin and four plumose setae apically; third article of palp with long setae, terminal article hooked, with a group of setae at hinge of unguis.

**Pereon.** Gnathopod I (Fig. 3A, B): coxal plate bearing a seta on anterior and posterior corners each; basis with long setae on anterior and posterior margins; carpus sub-parallel, 71% length of propodus, posterior margin with groups of setae; propodus pyriform, palm oblique, with one medial spine, seven spines on posterior margin and six spines on inner face; dactylus more than half of posterior margin in length, with one seta on outer margin.

Gnathopod II (Fig. 3C, D): coxal plate with two setae on anterior corner and one seta on posterior corner; basis with setae on anterior and posterior margins; carpus almost as long as propodus, with subparallel margins, bearing seven clusters of setae along ventral margin, a group of setae on dorsal margin; propodus subrectangular, palm transverse, with one medial spine and four spines on posterior corner; dactylus beyond the palm margin, with one seta on outer margin.

Pereopod III (Fig. 4F, G): coxal plate with two setae and one seta on anterior and posterior margins, respectively; basis with setae along anterior and posterior margins; merus with two spines on anterior margin and four clusters of setae on posterior margin, anterodistal corner with one spine accompanied by setae; carpus with three groups of spines accompanied by setae on posterior margin, anterodistal corner with one spine and posterodistal corner with two spines accompanied by setae; propodus with four groups of spines accompanied by setae on posterior margin, and two spines on posterodistal corner; dactylus stout, with one plumose seta on anterior margin and one seta at joint of unguis.

Pereopod IV (Fig. 4E, H): coxal plate concave posteriorly, with two setae on anterior corner and six setae on posterior margin; basis with one spine on anterodistal corner, and clusters of setae on posterior margin; merus with a spine on anterior margin, a spine accompanied with setae on anterodistal corner, three clusters of short setae on posterior margin; carpus and propodus with groups of spines accompanied by a few setae on posterior margins; dactylus with one plumose seta on anterior margin, and two setae at hinge of unguis.

Pereopod V (Fig. 5A): coxal plate with one seta on anterior lobe and four setae on posterior margin; basis posterior margin nearly straight, with four spines on anterior margin and one spine on anterodistal corner, and a row of 13 setae on posterior margin; merus with two spines on anterior margin and two spines on posterior margin, anterodistal and posterodistal corners with two spines each; carpus and propodus with groups of spines accompanied by fine setae on anterior margin, posterior margin of carpus with three groups of spines accompanied with setae; dactylus with two setae at hinge of unguis.

Pereopod VI (Fig. 5C, G): coxal plate with one seta and three setae on anterior and posterior margins, respectively; basis elongated, with one long seta and four spines on anterior margin, anterodistal corner with one spine accompanied by setae; posterior margin narrowing distally, with a row of 14 fine setae; merus with two groups of spines on anterior margin and two spines on posterior margin, anterodistal and posterodistal corners with two spines each; carpus and propodus with groups of spines on anterior margin, posterior margin of carpus with two pairs of spines accompanied by setae; dactylus with one seta at hinge of unguis.

Pereopod VII (Fig. 5B, H): coxal plate with five setae on posterior margin; basis expanded, with two long setae and four spines on anterior margin, anterodistal corner with two spines accompanied by setae, posterior margin with a row of 15 setae, and a spine on inner surface; merus with two pairs of spines on anterior margin and two single spines on posterior margin, anterodistal and posterodistal corners with two spines each; carpus and propodus with three or four pairs of spines on anterior margins, posterior margin of carpus with two pairs of spines, and posterior margin of propodus with two clusters of setae; dactylus with one plumose seta on posterior margin and two setae at hinge of unguis.

Coxal gills (Figs 3D, 4E, F, 5A–C): present on gnathopod II and pereopods III–VII, sac-like.

**Pleon.** Epimeral plates (Fig. 4C): plates I–III truncated to weakly acute, bearing two short setae on posterior margins. Plate I ventrally rounded, with nine setae on ventral margin; plate II with one spine on ventral margin; plate III with two spines on ventral margin.

Pleopods (Fig. 5D–F): subequal in length, peduncle with some marginal setae and two retinacula accompanied by two setae; inner and outer rami with approximately 20 segments, armed with plumose setae.

**Urosome.** Urosomites (Fig. 6E): non-humped, urosomites I–II bearing two-one-one-two spines on dorsal margin; urosomite III with two-one spines on lateral margins and two setae on dorsal margin.

Uropod I (Fig. 6C): peduncle longer than rami, with one basofacial spine, one spine on outer margin and two spines on inner margin, two spines and one spine on outer and inner distal corners respectively; inner ramus with one spine on inner margin, outer ramus with one spine on inner and outer margins each, both rami with five terminal spines.

Uropod II (Fig. 6A): peduncle with one spine on outer margin and one spine on outer distal corner; inner ramus with one spine on inner margin; outer ramus with one spine on outer margin, both rami with five terminal spines.

Uropod III (Fig. 6D): peduncle with one marginal spine and some setae; inner ramus length nearly one-third of outer ramus length, with three spines accompanied by setae on inner margin, outer margin bare; proximal article of outer ramus slender, with four pairs of spines on outer margin, six groups of spines and setae on inner margin, and four distal spines, terminal article cone-shaped, with simple setae distally; both rami with few short setae.

Telson (Fig. 6B): cleft, each lobe with three distal spines, one basolateral spine and some facial setae.

##### Description of paratype female.

(IZCAS-I-A0061-2), 10.1mm.

**Pereon.** Gnathopod I (Fig. 6F): propodus of gnathopod I ovate, palm slant, with seven spines on posterior corner, dactylus with one seta on outer margin.

Gnathopod II (Fig. 6G): propodus subrectangular, with two spines on posterior corner.

Oostegite: present on gnathopod II and pereopods III–V.

**Urosome.** Uropod III (Fig. 6H): inner ramus less than 30% length of outer ramus, with two spines on inner ramus and one distal spine accompanied by setae; outer ramus with four or five groups of spines accompanied by setae on inner and outer margins; both rami armed with few marginal setae.

##### Habitat.

This species was collected from a stream with altitude 4000 m, water clear and cold. There are a few water plants in the locality.

##### Remarks.

*Gammarus altus* sp. n. is most similar to *G. glaber* Hou, 2017 in having some setae on posterior margins of pereopods III and IV , and uropod III inner ramus length around one-third of outer ramus length, both rami with spines but few setae. The new species can be distinguished from *G. glaber* by the following characters (*G. glaber* in parentheses): second article of palp with two groups of B-setae (a group of B-setae); bases of pereopods V–VII elongated (broad), narrowing distally (posterior margin of pereopods V and VI nearly straight); uropod III with spines accompanied by simple setae on inner margin (uropod III with spines accompanied by plumose setae); and telson with no medial spines on surface (with one or two spines on medial surface).

This species is similar to *G. sichuanensis* Hou, Li & Zheng, 2002 in peduncle of antenna I and II with two or three groups of setae along anterior and posterior margins, and pereopod IV with a few setae on posterior margin. It differs from *G. sichuanensis* (*G. sichuanensis* in parentheses) by pereopod III with short setae on posterior margins of merus and carpus (with long setae on posterior margins of merus and carpus); bases of pereopods V–VII elongated (broad in *G. sichuanensis*); uropod III with no plumose setae (both rami with plumose setae on inner and outer margins).

#### Gammarus kangdingensis sp. n.

Taxon classificationAnimaliaORDOFAMILIA

http://zoobank.org/2883210F-54D8-4945-95C0-9C9DEE5D0DA2

**Figure 7.**
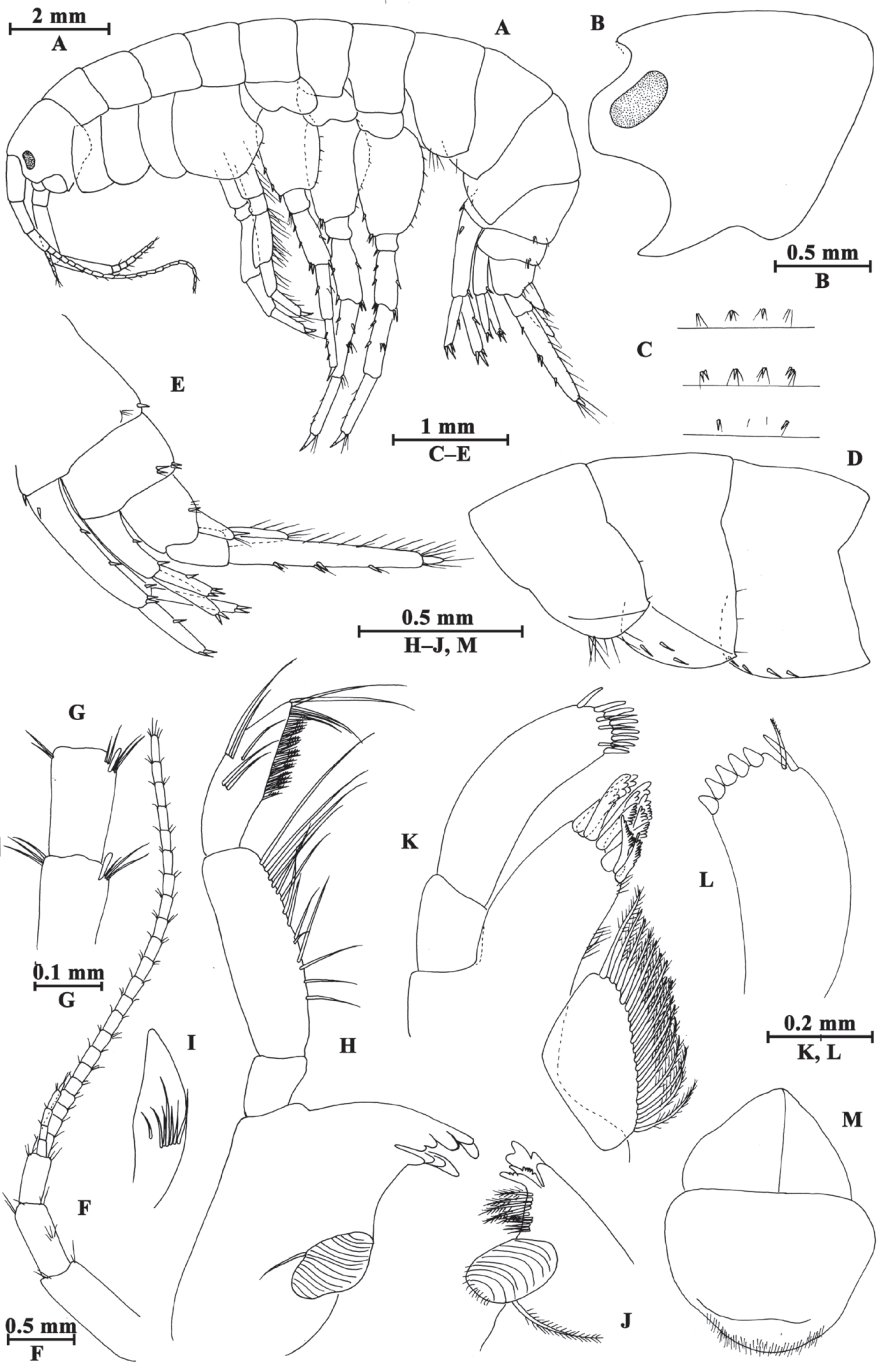
*Gammarus kangdingensis* sp. n., male holotype. **A** body (lateral view) **B** head **C** urosomites (dorsal view) **D** epimeral plates I–III **E** urosomites (lateral view) **F** antenna I **G** flagellum of antenna I **H** left mandible **I** inner face of article III of right palp **J** incisor of right palp **K** left maxilla **L** palp of right maxilla **M** upper lip.

**Figure 8.**
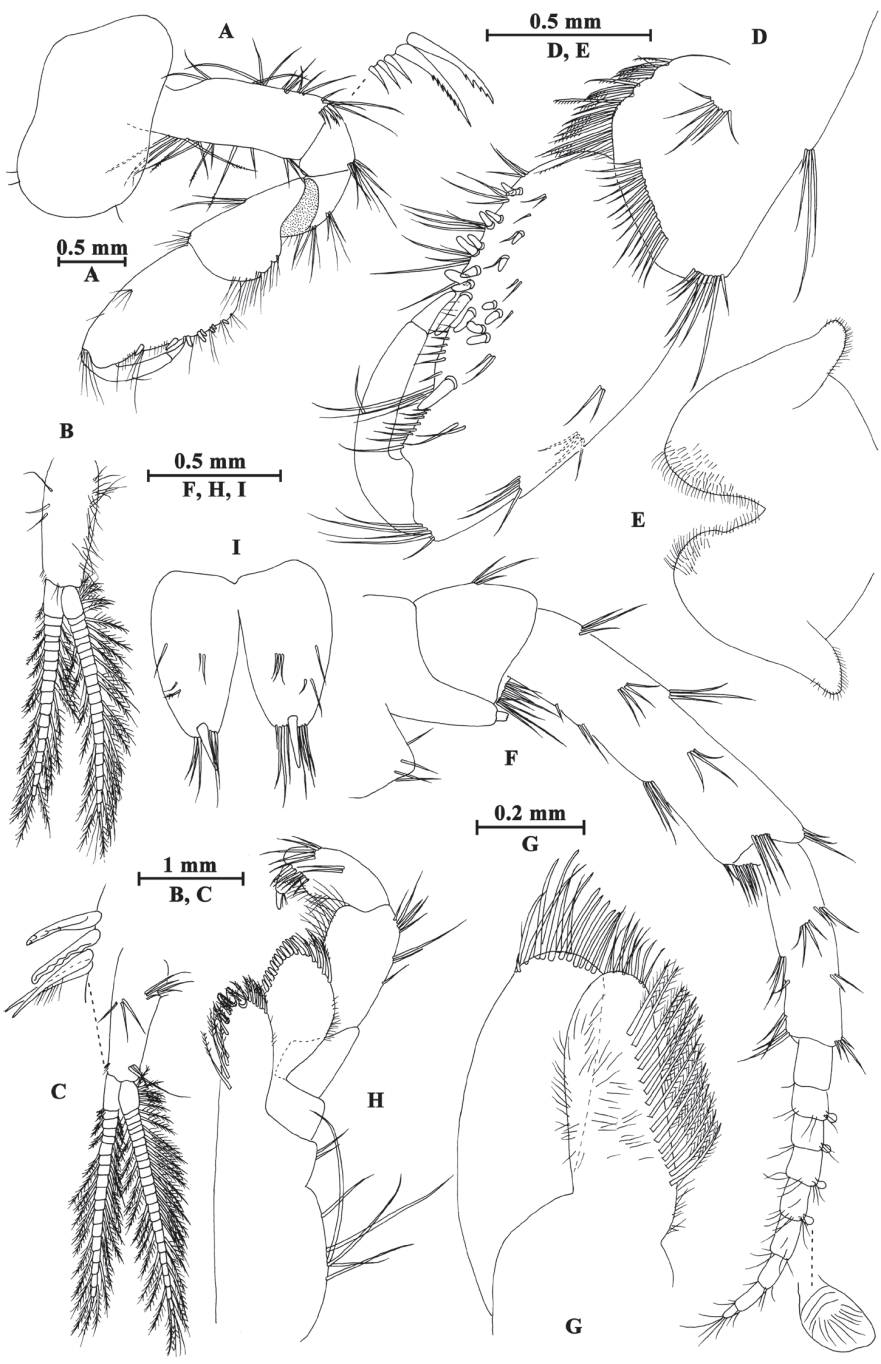
*Gammarus kangdingensis* sp. n., male holotype. **A** gnathopod I **B** pleopod II **C** pleopod I **D** propodus of gnathopod I (medial view) **E** lower lip **F** antenna II **G** maxilla II **H** maxilliped **I** telson.

**Figure 9.**
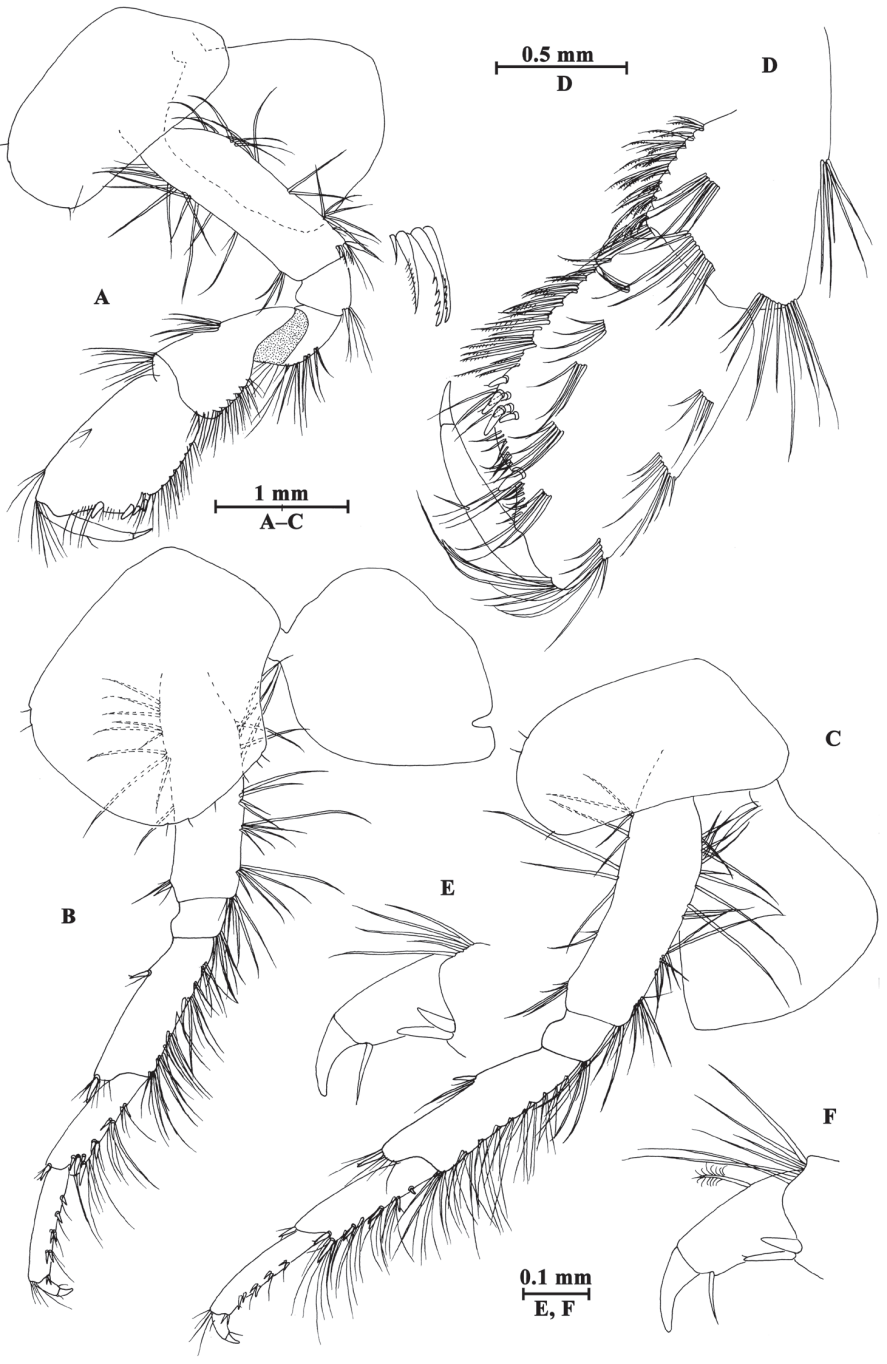
*Gammarus kangdingensis* sp. n., male holotype. **A** gnathopod II **B** pereopod IV **C** pereopod III **D** propodus of gnathopod (medial view) II **E** dactylus of pereopod IV **F** dactylus of pereopod III.

**Figure 10.**
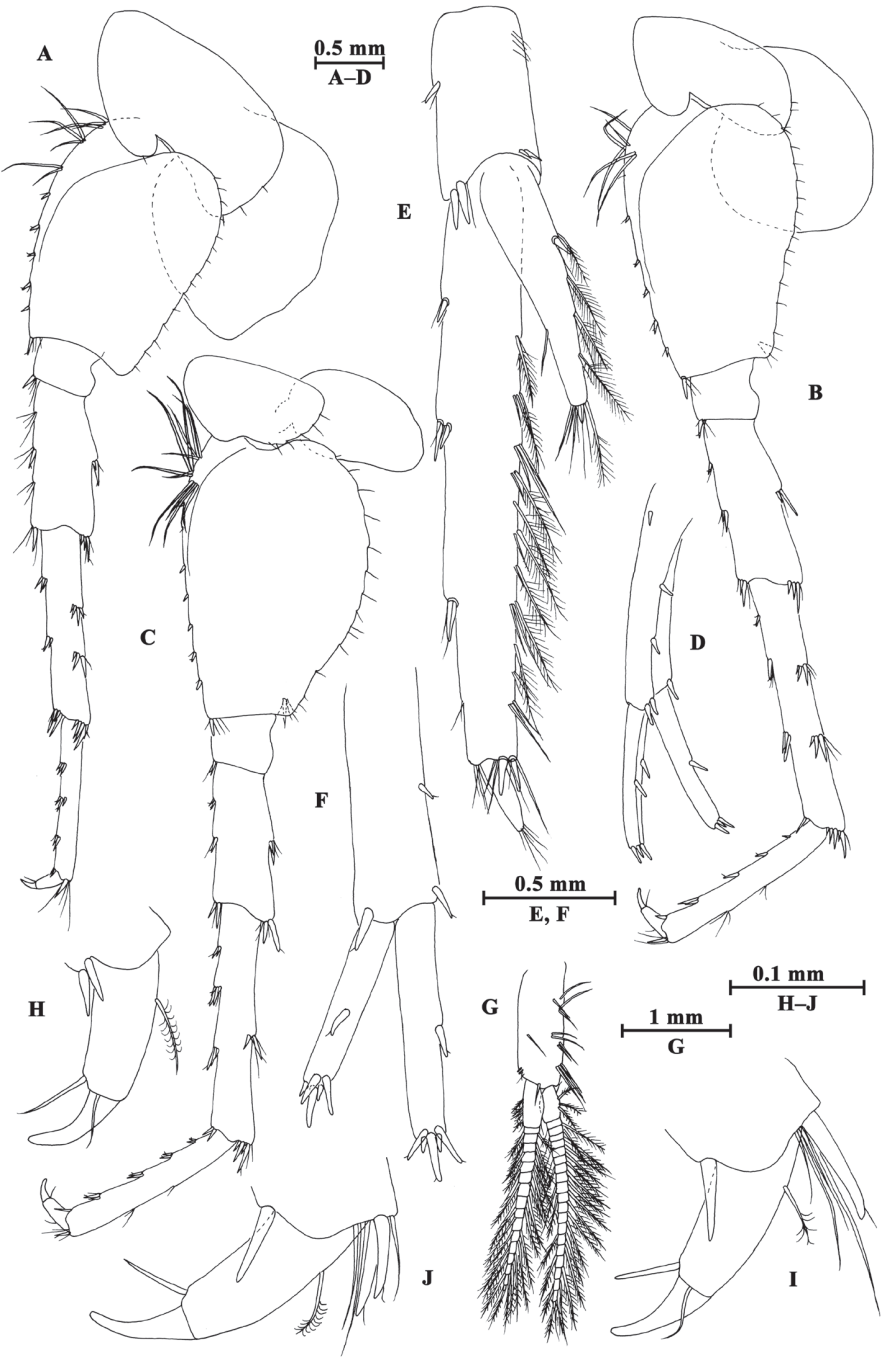
*Gammarus kangdingensis* sp. n., male holotype. **A** pereopod V **B** pereopod VI **C** pereopod VII **D** uropod I **E** uropod III **F** uropod II **G** pleopod III **H** dactylus of pereopod V **I** dactylus of pereopod VI **J** dactylus of pereopod VII.

**Figure 11.**
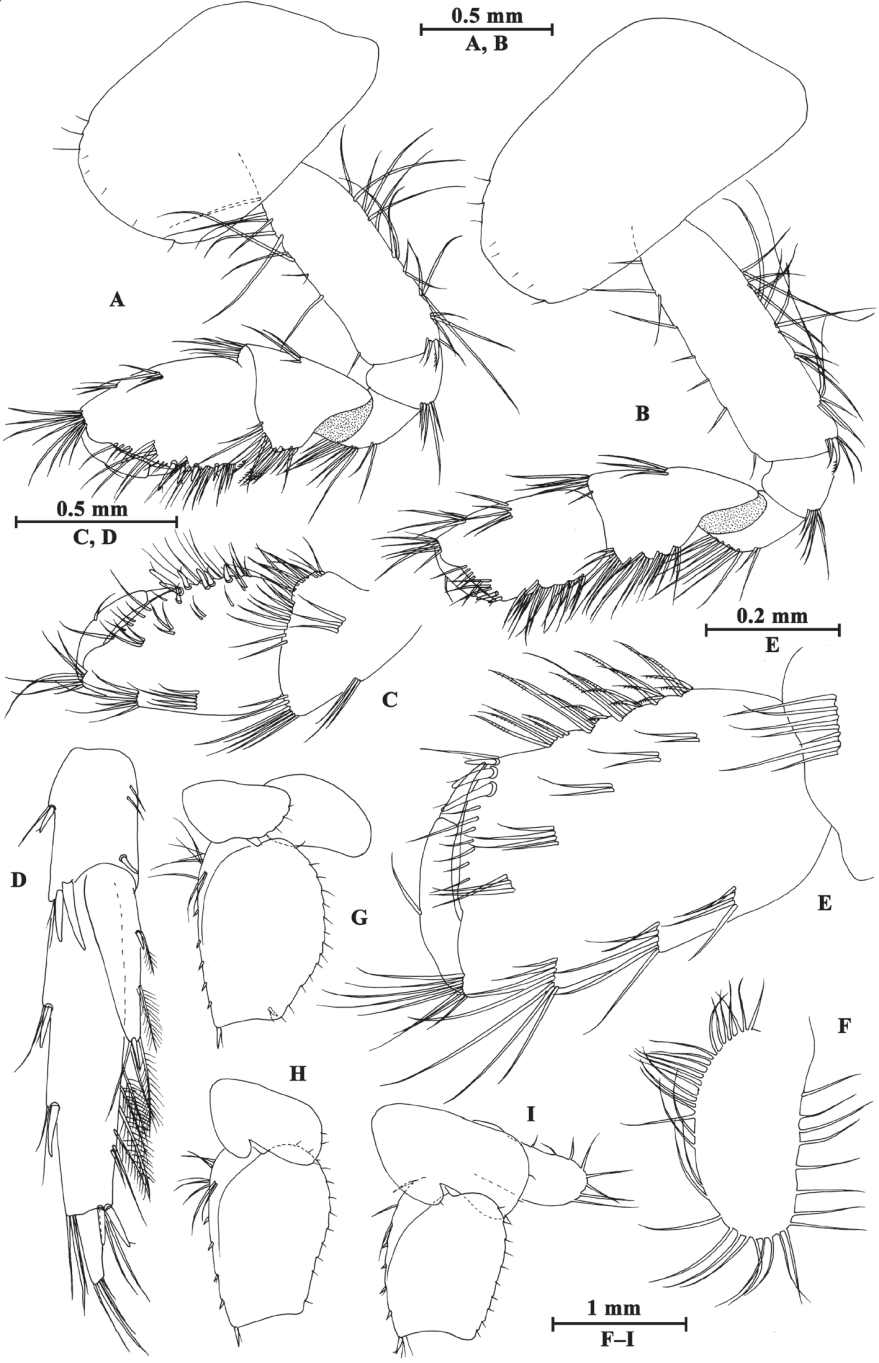
*Gammarus kangdingensis* sp. n., female paratype. **A** gnathopod I **B** gnathopod II **C** propodus of gnathopod I (medial view) **D** uropod III **E** propodus of gnathopod II (medial view) **F** oostegite of gnathopod II **G** basis of pereopod VII **H** basis of pereopod VI **I** basis of pereopod V.

Figs 7 , 8 , 9 , 10 , 11

##### Material examined.

Holotype: male (IZCAS-I-A0059-1), 11.8 mm, Erdaohe, Kangding County (30.0°N, 101.9°E), altitude 2470 m, August 19, 2001, collected by Xianjin Peng. Paratype: female (IZCAS-I-A0059-2), 8.5 mm; paratypes: two males and three females (IZCAS-I-A0059-3), same data as holotype.

##### Etymology.

The specific name is derived from the type locality; adjective.

##### Diagnosis.

Pereopods III and IV with long setae on posterior margins; epimeral plates blunt; uropod III inner ramus length less than half of outer ramus length, outer margin of outer ramus with few plumose setae.

##### Description of male holotype.

(IZCAS-I-A0059-1), 11.8 mm, slender.

**Head.** (Fig. 7A, B): cephalic lateral lobe truncated, inferior antennal sinus deep, eyes ovate.

Antenna I (Fig. 7F, G): peduncle articles I–III in length ratio 1.0 : 0.7 : 0.4, each article with distal setae; primary flagellum with 22 articles, most with aesthetascs; accessory flagellum with five articles.

Antenna II (Fig. 8F): gland cone reaching peduncle article III, peduncle articles VI and V in length ratio 1.0 : 0.7, with one or two groups of setae on ventral margin, flagellum with nine articles, each with short setae, calceoli present on articles II–V.

Upper lip (Fig. 7M): convex, with minute setae.

Mandible (Fig. 7H–J): incisor of left mandible with five teeth; lacinia mobilis with four teeth; second article of palp with 14 stiff setae, third article 77% the length of second article, with three groups of A-setae on outer face, seven B-setae on inner face, a row of 25 D-setae and five E-setae apically. Incisor of right mandible with four teeth; lacinia mobilis bifurcate, with small teeth.

Lower lip (Fig. 8E): inner lobes lacking, outer lobes covered with thin setae.

Maxilla I (Fig. 7K, L): asymmetrical, left inner plate with 18 plumose setae, outer plate with eleven serrated spines, second article of left palp with eight slender spines and three stiff setae; second article of right palp with five blunt spines and two stiff setae.

Maxilla II (Fig. 8G): inner plate with 15 plumose setae in an oblique row, outer plate broad, with apical setae.

Maxilliped (Fig. 8H): inner plate with three blunt spines and one subapical spine; outer plate broad, with 13 blade spines on medial margin and four plumose setae apically; palp with four articles, terminal article unguis-form, with setae at hinge of unguis.

**Pereon.** Gnathopod I (Fig. 8A, D): coxal plate weakly dilated distally, with two and one seta on anterior and posterior corners, respectively; basis with long setae along anterior and posterior margins; carpus a little shorter than propodus, with four groups of setae on posterior margin; propodus pyriform, palm oblique, bearing a median spine, ten spines on posterior margin and seven spines on inner face; dactylus reaching half of posterior margin of propodus, with one seta on outer margin.

Gnathopod II (Fig. 9A, D), coxal plate subrectangular, with one seta on anterior corner and one seta on posterior corner, basis slender than that of gnathopod I, bearing four serrated setae distally; carpus as long as propodus, with subparallel margins; propodus subrectangular, palm with a median blunt spine, and six spines on posterior corner; dactylus beyond the palm margin, with one seta on posterior margin.

Pereopod III (Fig. 9C, F): coxal plate with two setae on anterior corner and one seta on posterior corner; basis with long straight setae on posterior margin, merus with one spine accompanied by setae on anterior margin, and long setae on posterior margin; carpus with four groups of spines accompanied by long setae on posterior margin; propodus with four pairs of spines accompanied by few setae on posterior margin; dactylus short, with one plumose seta on anterior margin and one stiff seta at hinge of unguis.

Pereopod IV (Fig. 9B, E): shorter than pereopod III, coxal plate concave, with two setae on anterior corner and six setae on posterior margin; basis with long straight setae on posterior margin; merus with one spine accompanied by one seta on anterior margin and six clusters of long setae on posterior margin; carpus with three pairs of spines accompanied by long setae on posterior margin, posterodistal corner with two spines accompanied by long setae; propodus with four groups of spines accompanied by a few setae on posterior margin; dactylus with one seta at hinge of unguis.

Pereopod V (Fig. 10A, H): coxal plate with one seta on anterior lobe and three setae on posterior lobe; basis weakly expanded, with three groups of long setae and a row of five spines on anterior margin, anterodistal corner with a spine accompanied by setae, posterior margin with a row of 15 fine setae; merus with three groups of setae and spines on anterior margin, and a spine accompanied by setae on posterior margin; carpus with two groups of spines on anterior and posterior margins each; propodus with three groups of spines on anterior margin; dactylus with one plumose seta on posterior margin and two setae at hinge of unguis.

Pereopod VI (Fig. 10B, I): coxal plate with three setae on posterior margin; basis expanded, tapered distally, with six long setae and five spines on anterior margin, anterodistal corner with one spine accompanied with setae, posterior margin with 16 setae, and two setae on inner surface; merus and carpus with spines on anterior and posterior margins; propodus with four spines on anterior margin; dactylus with one plumose seta on posterior margin and two setae at hinge of unguis.

Pereopod VII (Fig. 10C, J): coxal plate with five setae on posterior margin; basis expanded, anterior margin with three groups of long setae and five spines, anterodistal corner with a spine accompanied by setae, posterior margin with a row of 15 setae, inner surface with a spine accompanied by setae; merus and carpus with spines on anterior and posterior margins; propodus with four pairs of spines on anterior margin; dactylus with one plumose seta on posterior margin and a seta at hinge of unguis.

Coxal gills (Figs 9A–C, 10A–C): present on gnathopod II and pereopods III–VII ovate, gill of pereopod VII smallest.

**Pleon.** Epimeral plates (Fig. 7D): plate I ventrally rounded, with nine setae on anteroventral margin and two setae on posterior margin; plate II with three spines on ventral margin and three setae on posterior margin, posterodistal corner blunt; plate III with four spines on ventral margin, posterodistal corner weakly acute.

Pleopods I–III (Figs 8B, C, 10G): subequal, peduncle with several marginal setae and two retinacula accompanied by two or three setae; inner and outer rami with approximately 23 articles, fringed with plumose setae.

**Urosome.** Urosomites I–III (Fig. 7C, E): non-humped, urosomites I–II with four groups of spines accompanied by setae; urosomite III with two dorsal setae and one spine accompanied by setae on each side.

Uropod I (Fig. 10D): peduncle with one basofacial spine, two spines on outer margin, two spines on outer distal corner, one spine on inner distal corner; outer ramus with one spine on each side; inner ramus with one mid-lateral spine on outer margin.

Uropod II (Fig. 10F): peduncle with one spine on inner margin, one spine on inner and outer distal corners each; outer ramus a little shorter than inner ramus, with one spine on outer margin; inner ramus with one spine on inner margin, both rami with five distal spines.

Uropod III (Fig. 10E), peduncle with one marginal spine and four distal spines; inner ramus 0.4 times as long as outer ramus, with one lateral and one distal spines, inner margin with four plumose setae; proximal article of outer ramus only with three pairs of spines on outer margin, inner margin with ten plumose setae, terminal article longer than adjacent spines, with simple setae distally.

Telson (Fig. 8F): deeply cleft, each lobe with one distal spine accompanied by some setae, and some facial short setae.

##### Description of paratype female.

(IZCAS-I-A0059-2), 8.5 mm.

**Pereon.** Gnathopod I (Fig. 11A, C): carpus and propodus shorter than those of male, carpus triangular; palm of propodus not oblique as that of male, palm with seven spines on posterior margin; dactylus with one seta on outer margin.

Gnathopod II (Fig. 11B, E): carpus and propodus elongated, carpus parallel-sided; propodus subrectangular, palm with three spines on posterior corner.

Pereopods III–VII (Fig. 11G–I): similar to those of male.

Oostegites (Fig. 11F, I): present on gnathopod II and pereopods III–V, oostegite of gnathopod II broad, with marginal setae; oostegite of pereopod V smallest.

**Urosome.** Uropod III (Fig. 11D): inner ramus length approx. half of outer ramus length, inner margins of inner and outer rami with a few plumose setae.

##### Habitat.

This species was collected from a small stream at the foot of mountain, with weak water flow but many detritus.

##### Remarks.

*Gammarus kangdingensis* sp. n. resembles *G. emeiensis* Hou, Li & Koenemann, 2002 in pereopods III and IV having long setae on posterior margins; pereopods V–VII having marginal spines, but with few setae; and epimeral plates with blunt posterodistal corners. *Gammarus kangdingensis* sp. n. can be distinguished from *G. emeiensis* (*G. emeiensis* in parentheses) in antenna II peduncle having short setae along anterior and posterior margins, and calceoli (peduncle with long setae on anterior and posterior margins, calceoli absent); uropod III inner ramus 0.4 times the length of outer ramus (inner ramus 0.7 times the length of outer ramus); uropod III terminal article of outer ramus longer than adjacent spines (terminal article as long as adjacent spines).

*Gamamrus kangdingensis* sp. n. is similar to *G. altus* sp. n. in the shape of gnathopods I and II. It differs from *G. altus* sp. n. (*G. altus* in parentheses) in having pereopods III and IV with long setae on posterior margins (with a few short setae on posterior margins); and inner ramus of uropod III 0.4 times the length of outer ramus (0.3), inner margins of inner and outer rami with a row of plumose setae (with no plumose setae).

#### Gammarus gonggaensis sp. n.

Taxon classificationAnimaliaORDOFAMILIA

http://zoobank.org/ED25CC10-321D-44B1-98F3-E2E30CC99286

**Figure 12.**
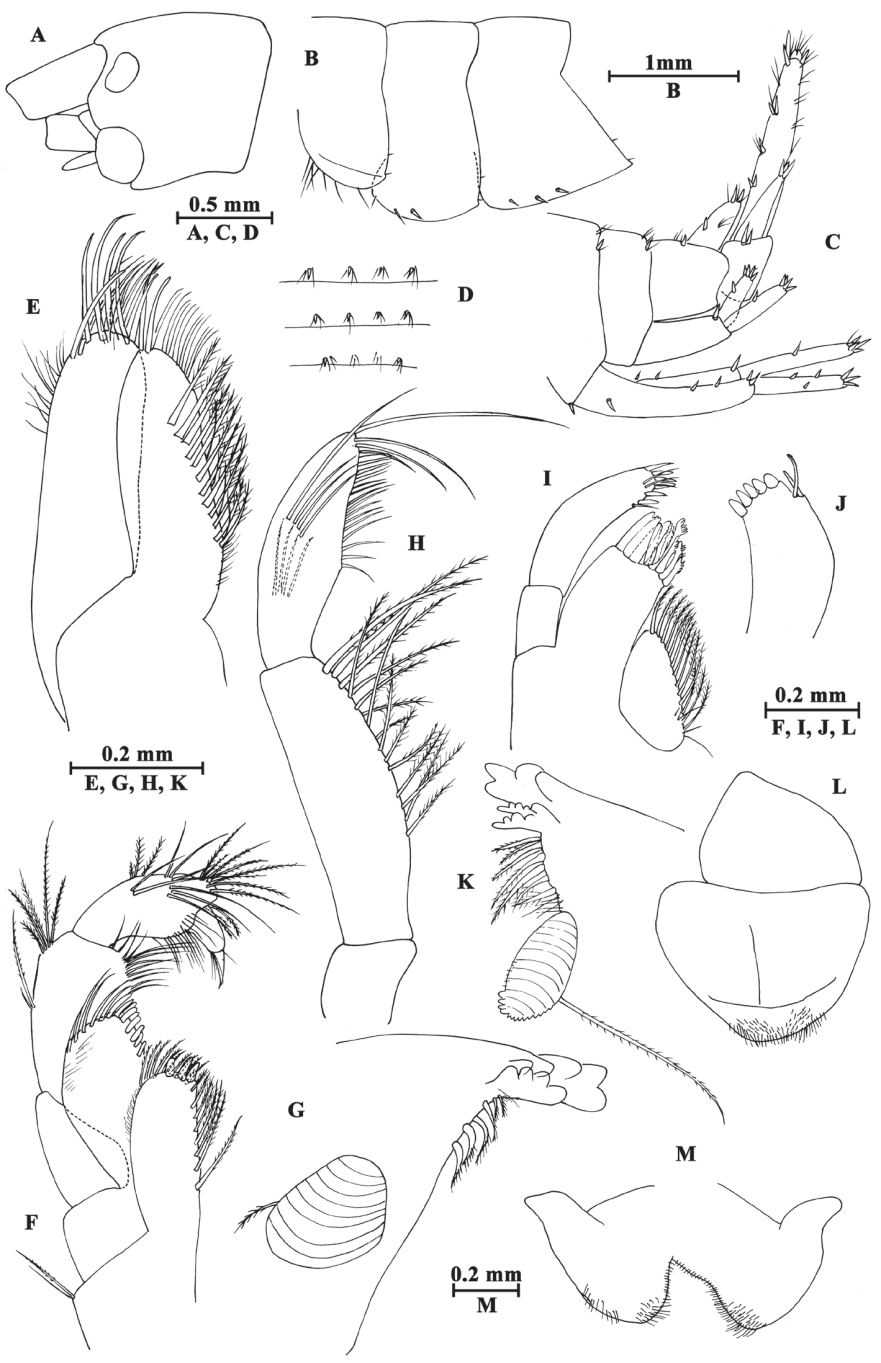
*Gammarus gonggaensis* sp. n., male holotype. **A** head **B** epimeral plates **C** urosomites (lateral view) **D** urosomites (dorsal view) **E** maxilla II **F** maxilliped **G** incisor of left mandible **H** palp of left mandible **I** left maxilla I **J** palp of right maxilla I **K** incisor of right mandible **L** upper lip **M** lower lip.

**Figure 13.**
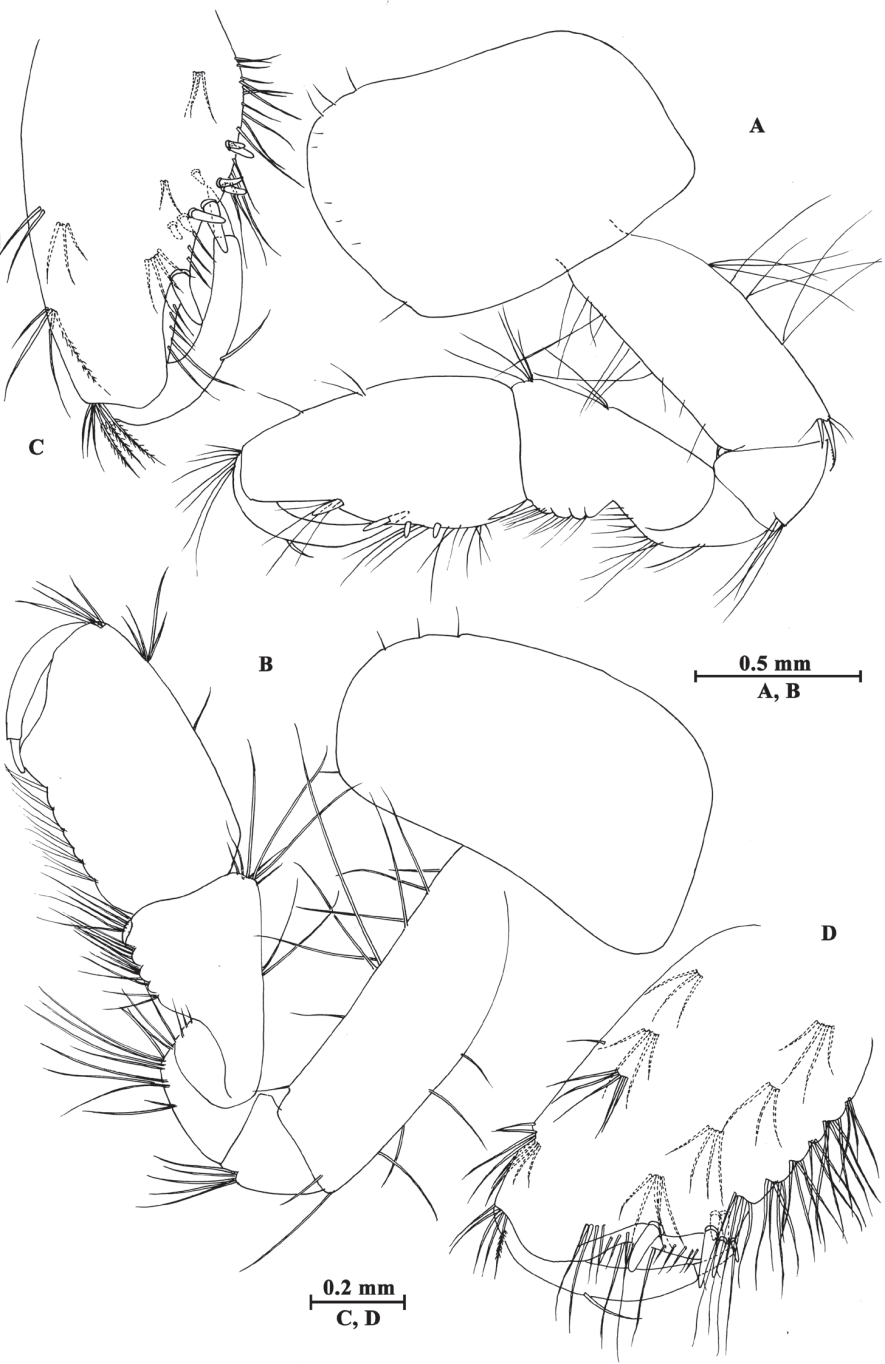
*Gammarus gonggaensis* sp. n., male holotype. **A** gnathopod I **B** gnathopod II **C** propodus of gnathopod I **D** propodus of gnathopod II.

**Figure 14.**
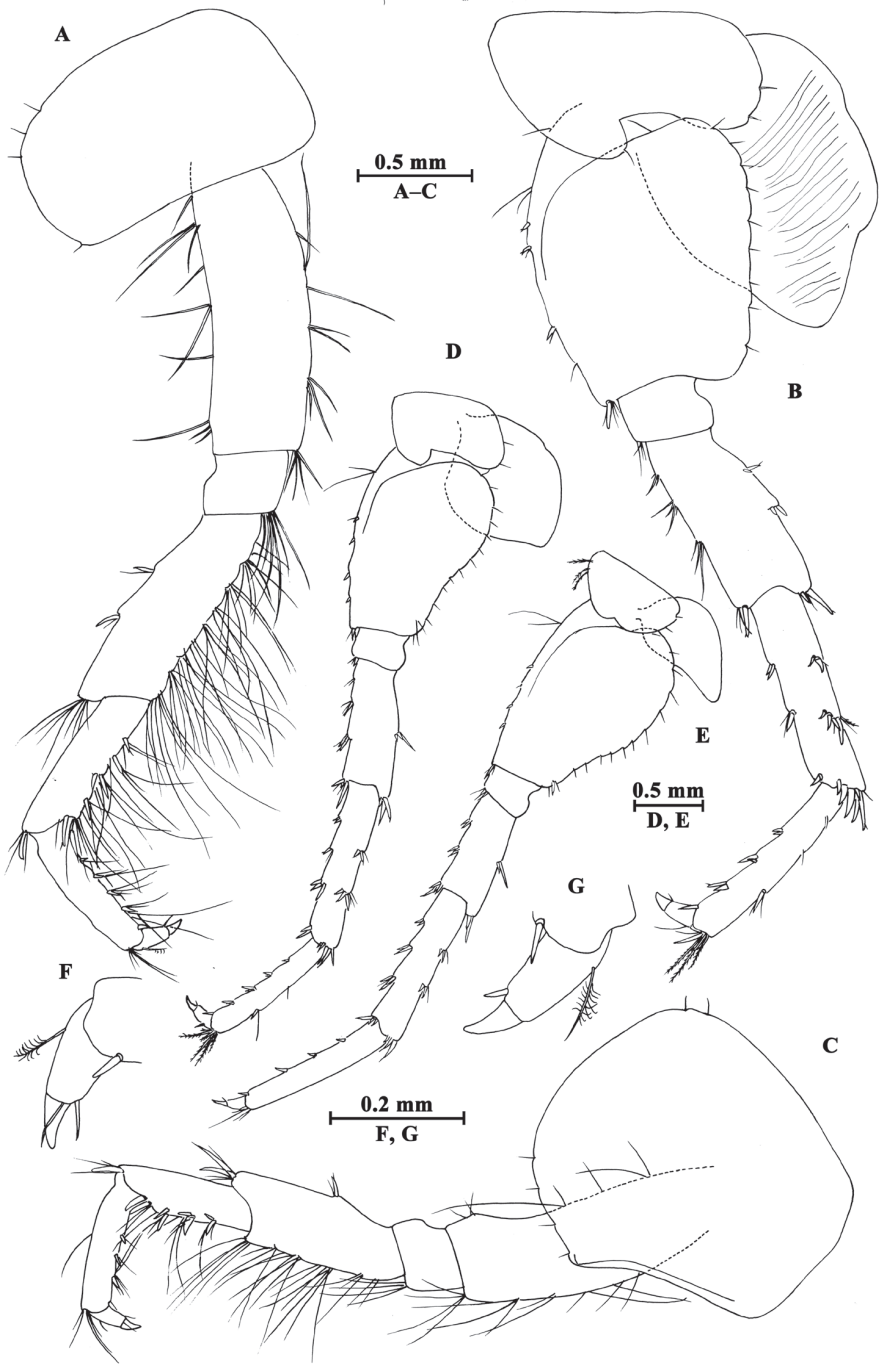
*Gammarus gonggaensis* sp. n., male holotype. **A** pereopod III **B** pereopod V **C** pereopod IV **D** pereopod VI **E** pereopod VII **F** dactylus of pereopod IV **G** dactylus of pereopod V.

**Figure 15.**
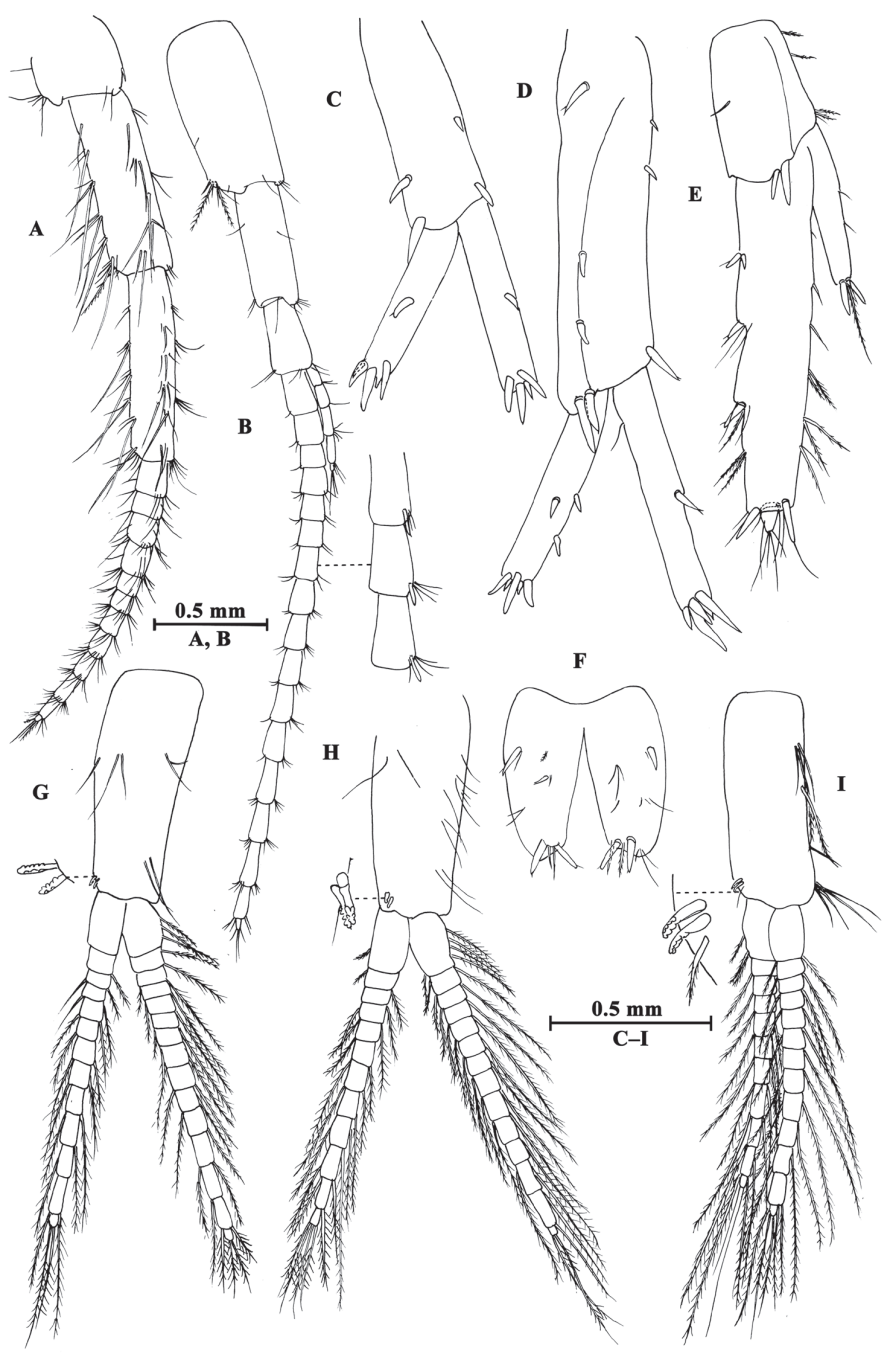
*Gammarus gonggaensis* sp. n., male holotype. **A** antenna II **B** antenna I **C** uropod II **D** uropod I **E** uropod III **F** telson **G** pleopod I **H** pleopod II **I** pleopod III.

**Figure 16.**
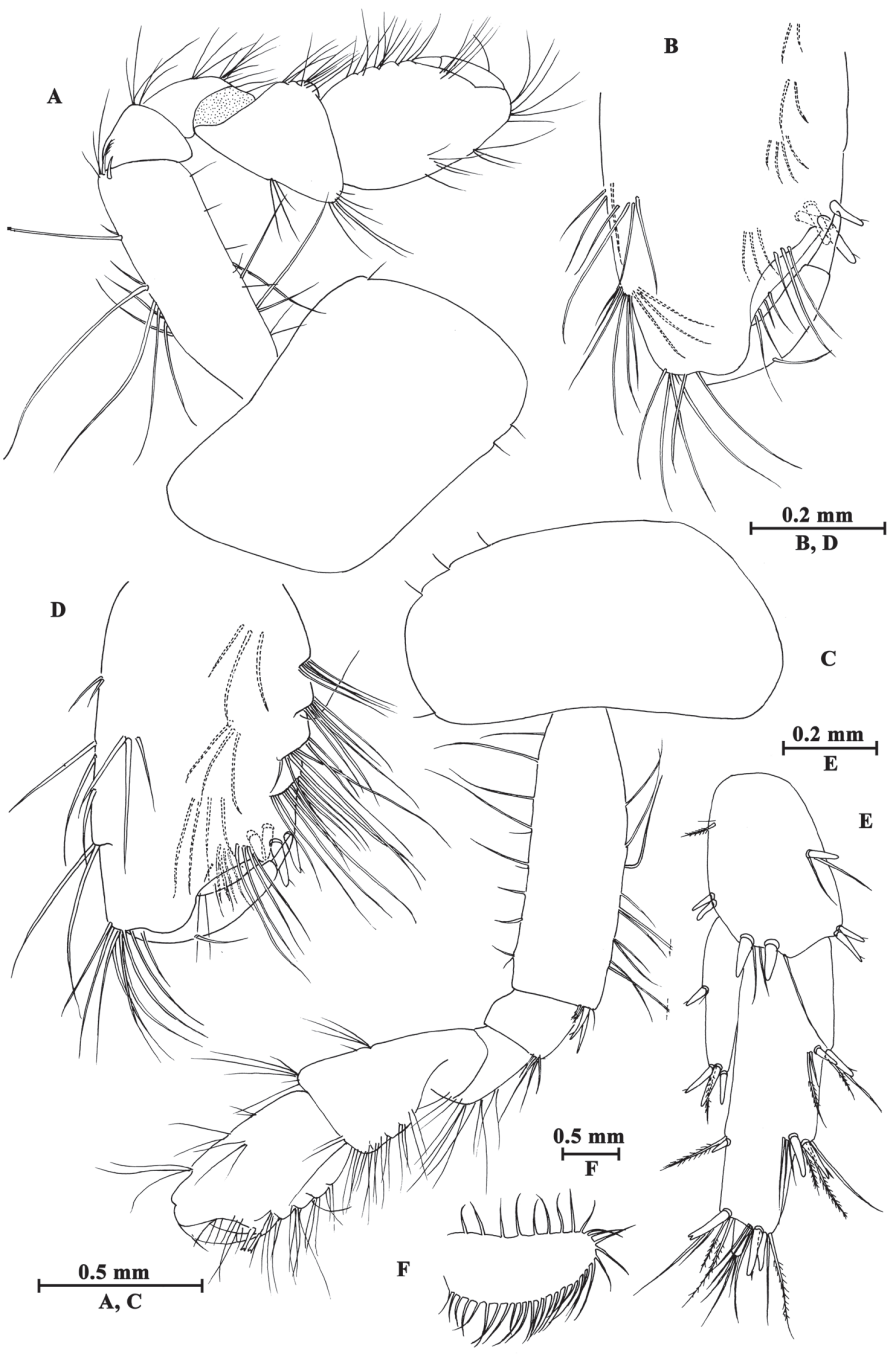
*Gammarus gonggaensis* sp. n., female paratype. **A** gnathopod I **B** propodus of gnathopod I **C** gnathopod II **D** propodus of gnathopod II **E** uropod III **F** oostegite of gnathopod II.

Figs 12 , 13 , 14 , 15 , 16

##### Material examined.

Holotype: male (IZCAS-I-A0065-1), 10.0 mm, Hepinggou, Baoxing County (30.3°N, 102.7°E), Gongga Mountains, altitude 2110 m, June 15, 2001, collected by Jinzhong Fu and Yuezhao Wang. Paratype: female (IZCAS-I-A0065-2) 8.6 mm; paratypes: two males (IZCAS-I-A0065-3), same data as holotype.

##### Etymology.

The specific name is derived from the type locality; adjective.

##### Diagnosis.

Antenna II with long setae along anterior and posterior margins of peduncle articles IV and V, calceoli absent; pereopods III and IV with long straight setae on posterior margin; inner ramus of uropod III less than half the length of outer ramus, both inner and outer rami with few marginal setae.

##### Description of holotype male.

(IZCAS-I-A0065-1), 10.0 mm.

**Head.** (Fig. 12A): Eyes medium in size, oval.

Antenna I (Fig. 15B): peduncle articles I–III in length ratio 1.0 : 0.7 : 0.5, bearing distal setae; flagellum with 17 articles, aesthetascs present; accessory flagellum with five articles.

Antenna II (Fig. 15A): gland cone reaching peduncle article III, peduncle article III with distal setae, article IV a little shorter than article V, both with five to six groups of long setae along anterior and posterior margins; flagellum with 12 articles, bearing some setae, calceoli absent.

Upper lip (Fig. 12L): convex, with minute setae.

Mandible (Fig. 12G, H, K): incisor of left mandible with five teeth; lacinia mobilis with four teeth; second and third articles of palp in length ratio 1.1 : 1.0, second article with 16 setae, third article with four A-setae, four B-setae, 19 D-setae and four E-setae apically. Incisor of right mandible with four teeth; lacinia mobilis bifurcate, with several small teeth.

Lower lip (Fig. 12M): inner lobes lacking, outer lobes covered with thin setae.

Maxilla I (Fig. 12I, J): asymmetrical, left inner plate with 13 plumose setae, outer plate with eleven serrated spines, second article of left palp bearing ten slender spines; second article of right palp with five blunt spines and two stiff setae.

Maxilla II (Fig. 12E): inner plate with 12 plumose setae in an oblique row; inner and outer plates with long setae apically.

Maxilliped (Fig. 12F): inner plate with three apical spines and several plumose setae; outer plate with nine blade spines on medial margin and five long setae apically; palp with four articles.

**Pereon.** Gnathopod I (Fig. 13A, C): coxal plate weakly dilated distally, bearing three and one seta on anterior and posterior corners respectively; basis with setae on anterior and posterior margins; carpus and propodus in length ratio 0.6 : 1.0; palm of propodus oblique, bearing one spine on medial margin, three pairs of spines on posterior margin and three spines on inner face; dactylus more than half of posterior margin in length, with on seta one outer margin.

Gnathopod II (Fig. 13B, D): coxal plate subrectangular, with three and one seta on anterior and posterior corners respectively; carpus parallel-sided; palm of propodus truncated, bearing one medial spine, five spines on posterior corner; dactylus beyond the palm margin, with one seta on outer margin.

Pereopod III (Fig. 14A): coxal plate with three and one seta on anterior and posterior corners respectively, merus to propodus with groups of long straight setae on posterior margins; carpus and propodus accompanied by five spines on posterior margins, dactylus stout.

Pereopod IV (Fig. 14C, F): coxal plate concave, bearing two and four setae on anterior corner and posterior margin, merus with six groups of long setae on posterior margin; carpus and propodus with four groups of spines accompanied by some setae; dactylus with one seta on anterior margin and two setae at hinge of nail.

Pereopod V (Fig. 14B, G): coxal plate bearing one seta on anterior lobe and two setae on posterior lobe; basis with three setae and three spines on anterior margin, anterodistal corner with one spine accompanied by setae, posterior margin with a row of nine setae; merus with three groups of setae on anterior margin, a spine accompanied by setae on anterodistal corner, two pairs of spines on posterior margin; carpus with two groups of spines on anterior and posterior margins each, anterodistal corner with a spine, posterodistal corner with a group of six spines; propodus with three pairs of spines on anterior margin, and three clusters of setae and spines on posterior margin; dactylus with one plumose seta on posterior margin and a seta at hinge of unguis.

Pereopod VI (Fig. 14D): coxal plate with two setae on posterior margin; basis narrowing distally, with two long setae and four spines on anterior margin, anterodistal corner with one spine accompanied by setae, posterior margin with a row of 13 setae, and inner surface with two setae on posterior corner; merus with three groups of spines on anterior margin and a pair of spines on posterior margin; carpus with three or two groups of spines on anterior and posterior margins; propodus with four pairs of spines on anterior margin, posterior margin with a seta and a spine accompanied by a seta; dactylus similar to that of pereopod V.

Pereopod VII (Fig. 14E): coxal plate with three plumose setae on anterior margin and three setae on posterior margin; basis with two setae and four spines on anterior margin, posterior margin with a row of 12 setae, inner surface with one spine accompanied by setae on posterior corner; merus and carpus with spines on anterior and posterior margins; propodus with three pairs of spines on anterior margin; dactylus similar to that of pereopod V.

Coxal gills (Fig. 14B, D, E): present on gnathopod II and pereopods III–VII sac-like.

**Pleon.** Epimeral plates (Fig. 12B): plate I ventrally rounded, with seven setae on ventral margin and two setae on posterior margin; plate II with one seta and two spines on anterior corner, two setae on posterior margin, posterodistal corner blunt; plate III with three spines on ventral margin and two setae on posterior margin.

Pleopods I–III (Fig. 15G–I): peduncle with some marginal setae, bearing two retinacula accompanied by setae; inner and outer rami nearly the same length, both rami fringed with plumose setae.

**Urosome.** Urosomites I–III (Fig. 12 C, D): non-humped, with four clusters of spines accompanied by setae.

Uropod I (Fig. 15D): peduncle with one basofacial spine, two spines on outer margin, two spines on outer distal corner, two spines on inner margin, and one spine on inner distal corner; outer ramus with one and two spines on outer and inner margins respectively; inner ramus with one spine on inner margin, both rami with five terminal spines.

Uropod II (Fig. 15C): peduncle with one spine on inner and outer margins each, one spine on inner and outer distal corners each; inner ramus with one spine on inner margin; outer ramus with one spine on outer margin, both rami with five terminal spines.

Uropod III (Fig. 15E): peduncle with one seta on surface, two marginal plumose setae, two distal plumose setae and two distal spines; inner ramus 0.4 times as long as outer ramus, bearing two distal spines accompanied by one long seta; proximal article of outer ramus with two-one-two spines on outer margin and two distal spines, inner margin with a few plumose setae, terminal article shorter than adjacent spines, with distal setae.

Telson (Fig. 15F): cleft, with two distal spines and one facial spine.

##### Description of paratype female.

(IZCAS-I-A0065-2), 8.6 mm.

**Pereon.** Gnathopod I (Fig. 16A, B): propodus pyriform, palm slant, bearing four spines on posterior corner.

Gnathopod II (Fig. 16C, D): palm of propodus transverse, with four spines on posterior corner.

Oostegites (Fig. 16F): present on gnathopod II and pereopods III–V, broad, with many long setae.

**Urosome.** Uropod III (Fig. 16E): peduncle with one marginal spine accompanied by setae, and three pairs of distal spines; inner ramus around half the length of outer ramus, bearing one marginal and three distal spines; proximal article of outer ramus with two groups of spines on outer margin, one spine on inner margin and three spines on distal margin, terminal article shorter than adjacent spines.

##### Habitat.

This species was collected from a small brook flowing under a large stone, forming a pool with an area of one square meter.

##### Remarks.

The new species of *Gammarus gonggaensis* sp. n. is similar to *G. kangdingensis* sp. n. in pereopods III and IV having long setae on posterior margins; and inner ramus of uropod III 0.4 times the length of outer ramus. *Gammarus gonggaensis* sp. n. can be distinguished from *G. kangdingensis* sp. n. (*G. kangdingensis* in parentheses) by antenna II peduncle having long setae along anterior and posterior margins, calceoli absent (peduncle with short setae, calceoli present); outer ramus of uropod III with a few plumose setae on inner margin, inner ramus with no plumose marginal setae (with a row of plumose setae on inner margins of inner and outer rami); terminal article of uropod III shorter than adjacent spines (longer than adjacent spines).

*Gammarus gonggaensis* sp. n. is similar to *G. emeiensis* Hou, Li & Koenemann, 2002 in antenna II peduncle with long setae along anterior and posterior margins, calceoli absent, and pereopods III and IV with long setae on posterior margins. It differs from *G. emeiensis* Hou et al., 2002 by uropod III inner ramus 0.4 times the length of outer ramus, while reaching 0.7 times in *G. emeiensis*.

*Gammarus gonggaensis* sp. n. differs from *G. altus* sp. n. (*G. altus* in parentheses) by anternna II peduncle with long setae, calceoli absent (with short setae, calceoli present); pereopods III and IV with long setae on posterior margin (with a few short setae); uropod III with plumose setae on inner margin of inner ramus (with no plumose setae), terminal article shorter than adjacent spines (longer than adjacent spines).

#### Gammarus limosus sp. n.

Taxon classificationAnimaliaORDOFAMILIA

http://zoobank.org/9ACD4C39-16EC-47AF-A3B0-C32C3BA8EAC3

**Figure 17.**
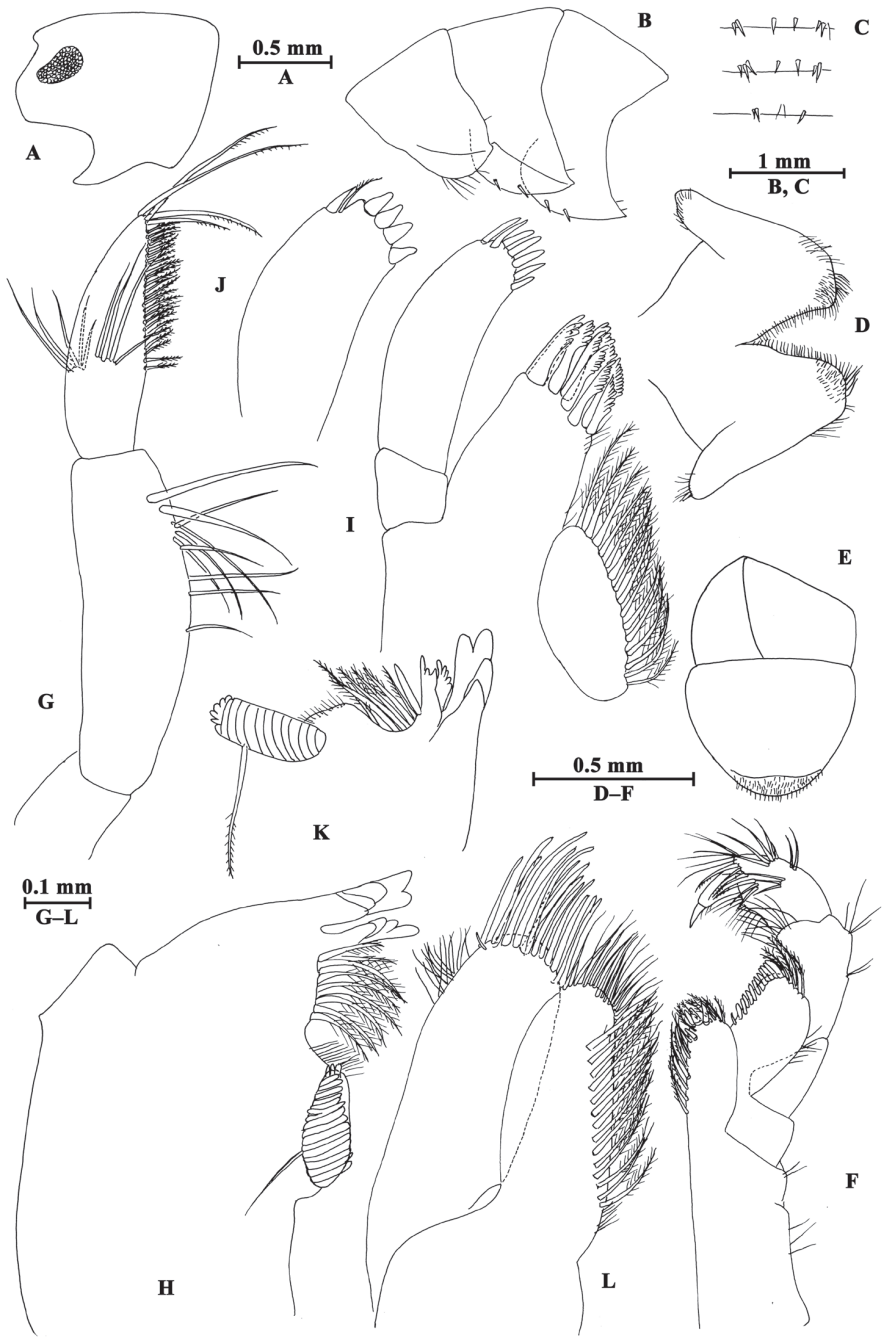
*Gammarus limosus* sp. n., male holotype. **A** head **B** epimeral plates I–III **C** urosomites I–III (dorsal view) **D** lower lip **E** upper lip **F** maxilliped **G** palp of left mandible **H** incisor of left mandible **I** left maxilla I **J** palp of right maxilla I **K** incisor of right mandible **L** maxilla II.

**Figure 18.**
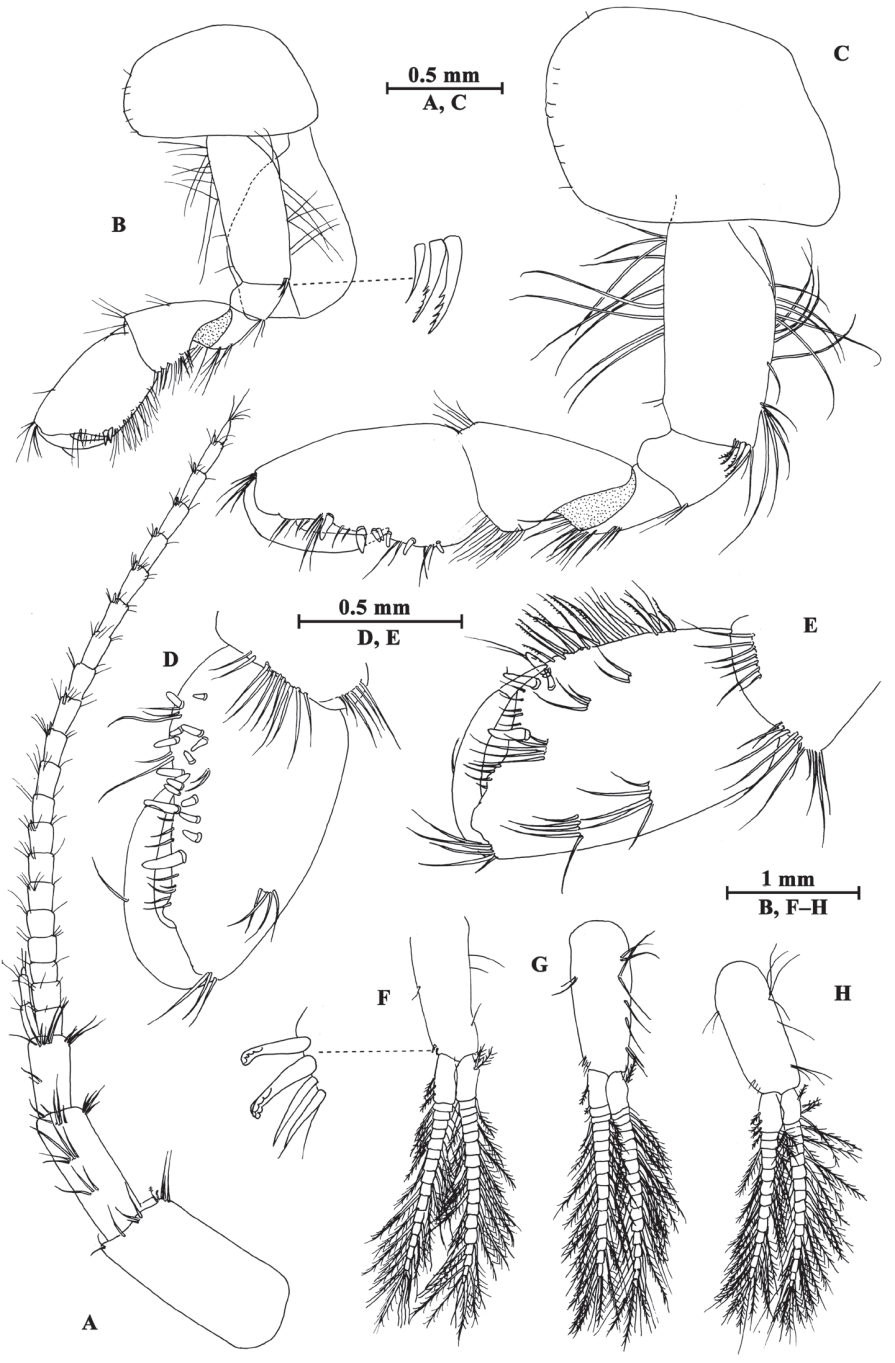
*Gammarus limosus* sp. n., male holotype. **A** antenna I **B** gnathopod II **C** gnathopod I **D** propodus of gnathopod I (medial view) **E** propodus of gnathopod II (medial view) **F** pleopod I **G** pleopod II **H** pleopod III.

**Figure 19.**
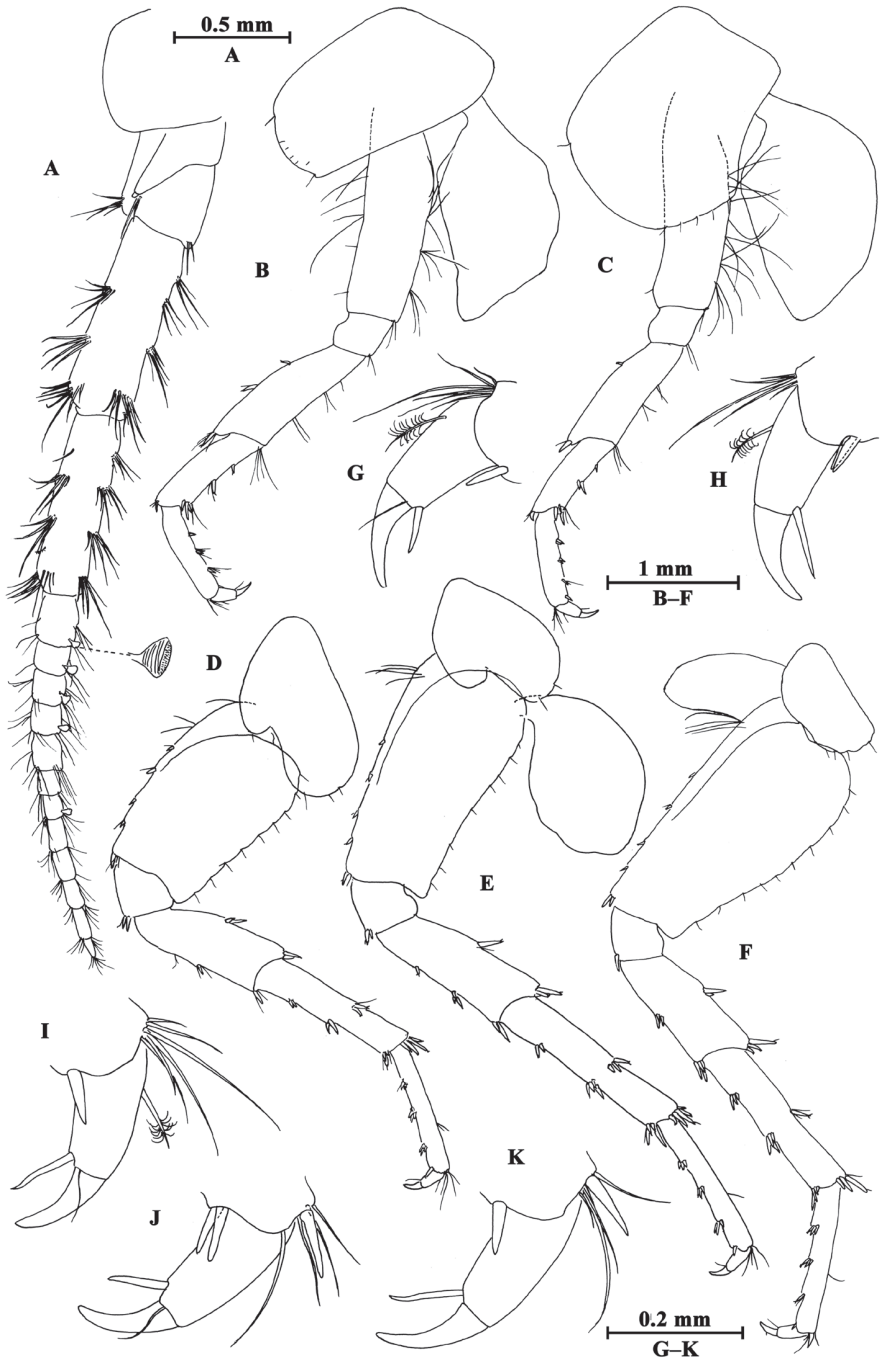
*Gammarus limosus* sp. n., male holotype. **A** antenna II **B** pereopod III **C** pereopod IV **D** pereopod V **E** pereopod VI **F** pereopod VII **G** dactylus of pereopod III **H** dactylus of pereopod IV **I** dactylus of pereopod V **J** dactylus of pereopod VI **K** dactylus of pereopod VII.

**Figure 20.**
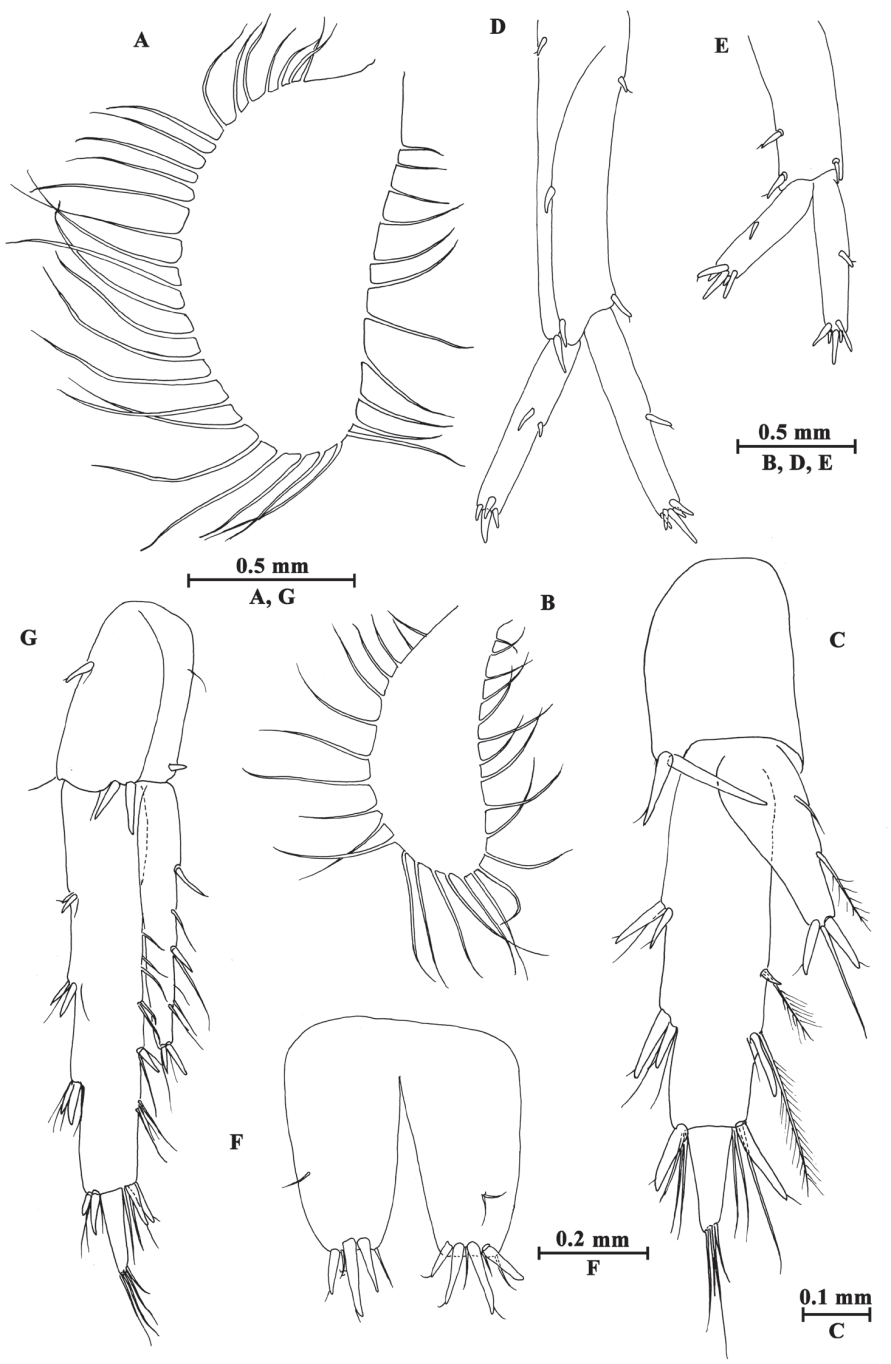
*Gammarus limosus* sp. n., **D–G** male, holotype; **A–C** female, paratype. **A** oostegite of gnathopod II **B** oostegite of pereopod III **C** uropod III **D** uropod I **E** uropod II **F** telson **G** uropod III.

**Figure 21.**
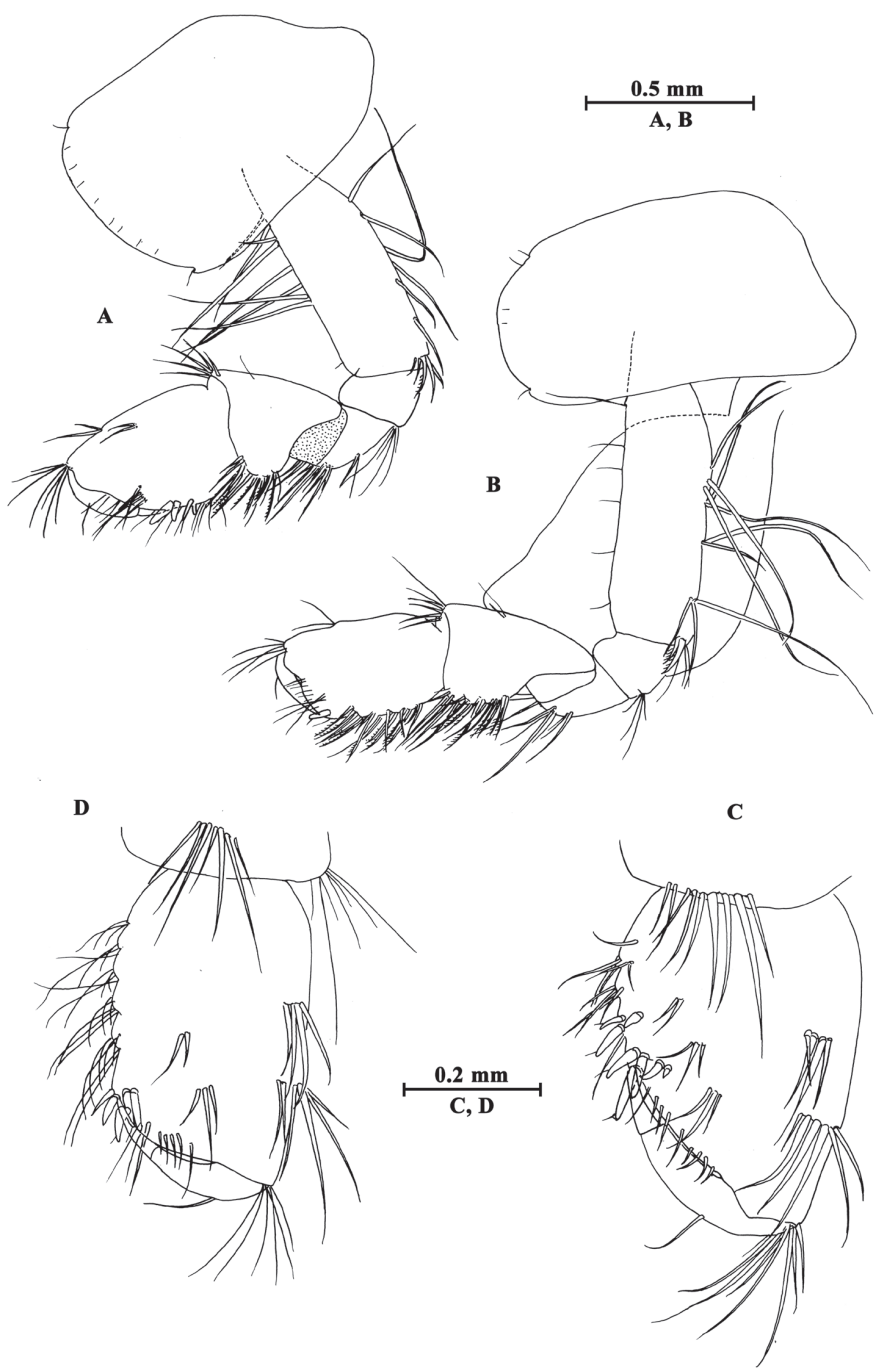
*Gammarus limosus* sp. n., female paratype. **A** gnathopod I **B** gnathopod II **C** propodus of gnathopod I (medial view) **D** propodus of gnathopod II (medial view).

**Figure 22.**
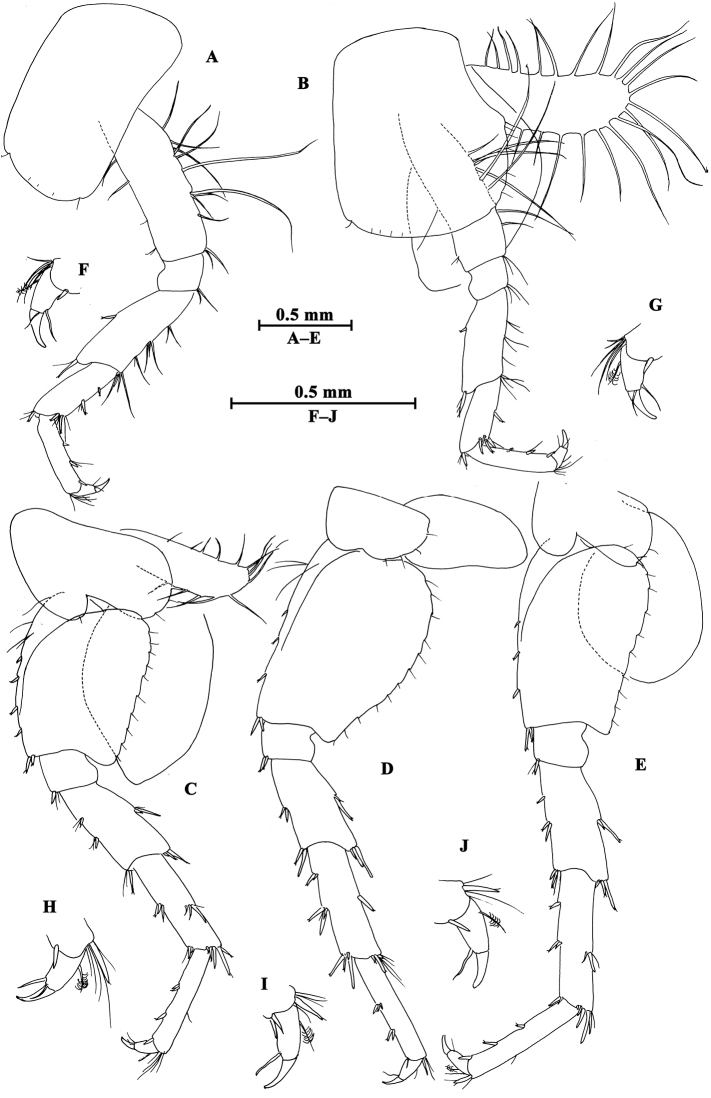
*Gammarus limosus* sp. n., female paratype. **A** pereopod III **B** pereopod IV **C** pereopod V **D** pereopod VII **E** pereopod VI **F** dactylus of pereopod III **G** dactylus of pereopod IV **H** dactylus of pereopod V **I** dactylus of pereopod VII **J** dactylus of pereopod VI.

Figs 17 , 18 , 19 , 20 , 21 , 22

##### Material examined.

Holotype: male (IZCAS-I-A0063-1), 8.6 mm, Baxoi County (30.2°N, 97.2°E), Tibet, altitude 4400 m, August 21, 2001, collected by Xianjin Peng. Paratype: female (IZCAS-I-A0063-2), 5.5 mm; paratypes: five males and five females (IZCAS-I-A0063-3), same data as holotype.

##### Etymology.

This specific name alludes to its living environment, living silt beside a polluted river; adjective.

**Diagnosis.** Antenna I accessory flagellum with three articles; antenna II calceoli present; pereopods III and IV with few setae on posterior margin; inner ramus of uropod III reaching 0.6 times the length of outer ramus, terminal article of outer ramus longer than adjacent spines, both inner and outer rami with few marginal setae.

##### Description of holotype male.

(IZCAS-I-A0063-1), 8.6 mm.

**Head.** (Fig. 17A): cephalic lateral lobe truncated, inferior antennal sinus deep, eyes ovate.

Antenna I (Fig. 18A): peduncle articles I–III in length ratio 1.0 : 0.7: 0.4, each article with distal setae, article II with two groups of setae on inner face; primary flagellum with 21 articles, most with aesthetascs; accessory flagellum with three articles, the distal article tiny.

Antenna II (Fig. 19A): gland cone shorter than peduncle article III, article IV approximately as long as article V, both with two or three groups of short setae along anterior and posterior margins; flagellum with 12 articles, proximal six articles with calceoli.

Upper lip (Fig. 17E): convex, with minute setae.

Mandible (Fig. 17G, H, K): asymmetrical, left incisor with five teeth; lacinia mobilis with four teeth; spine row with nine plumose setae; second article of palp with ten setae on medial margin, third article 0.7 times the length of second article, with four A-setae on outer face, five B-setae on inner face, a row of 22 D-setae and four E-setae. Incisor of right mandible with four teeth; lacinia molibis bifurcate, with small teeth; molar with one long seta.

Lower lip (Fig. 17D): inner lobes lacking, outer lobes covered with thin setae.

Maxilla I (Fig. 17I, J): asymmetrical, left inner plate with 15 plumose setae on medial margin; outer plate with eleven serrated spines; second article of palp with seven slender spines. Second article of right palp broad, with five blunt spines and one stiff seta.

Maxilla II (Fig. 17L): inner plate with 14 plumose setae on inner face; inner and outer plates with apical setae.

Maxilliped (Fig. 17F): inner plate with three apical and one subapical spines, and 17 plumose setae; outer plate with 13 blade spines on medial margin and five apical plumose setae; palp with four articles, terminal article unguis-form.

**Pereon.** Gnathopod I (Fig. 18C, D): coxal plate weakly dilated distally, lower margin with short setae; basis with long setae on anterior and posterior margins; carpus triangular, posterior margin with setae; propodus oval, palm oblique, with one median spine, seven spines on posterior margin and seven spines on inner face; dactylus longer than half of posterior margin, with one seta on outer margin.

Gnathopod II (Fig. 18B, E): coxal plate slightly narrowing distally, with short setae on lower margin; basis with long setae on anterior and posterior margins; carpus a little shorter than propodus, with sub-parallel margins, bearing setae on posterior margin; propodus subrectangular, palm transverse, with one median spine and four spines on posterior corner; dactylus beyond the palm margin, with one seta on outer margin.

Pereopod III (Fig. 19B, G): coxal plate with one seta on anterodistal and posterodistal corners each; basis with long setae on anterior and posterior margins; merus to propodus with few setae on posterior margin; merus with two spines on anterior margin and three clusters of setae on posterior margins; carpus and propodus with three groups of spines accompanied by setae on posterior margins; dactylus with one plumose seta on anterior margin and one simple and one stiff setae at hinge of unguis.

Pereopod IV (Fig. 19C, H): coxal plate concave, with one seta on anterior corner and five setae on posterior margin; basis with long setae on posterior margin; merus with one spine on anterior margin, posterior margin with two pairs of setae; carpus and propodus with two to four groups of spines accompanied by setae on posterior margins; dactylus with one plumose seta on anterior margin and a seta at hinge of unguis.

Pereopod V (Fig. 19D, I): coxal plate with one seta on anterior lobe and three setae on posterior lobe; basis nearly straight on posterior margin, with three setae and four spines on anterior margin, anterodistal corner with two spines accompanied by setae; posterior margin with a row of nine setae; merus and carpus with two groups of spines accompanied with few setae on anterior margins and one group of spines on posterior margins; propodus with three groups of spines on anterior margin and a pair of setae on posterior margin; dactylus with one plumose seta on posterior margin and two setae at hinge of unguis.

Pereopod VI (Fig. 19E, J): coxal plate with two setae on posterior margin; basis elongated, anterior margin with four setae and four spines on anterior margin, anterodistal corner with one spine, posterior margin with a row of eight setae; merus and carpus with two groups of spines on anterior margins and a group of spines on posterior margins; propodus with three groups of spines on anterior margin and a seta on posterior margin; dactylus with two setae at hinge of unguis.

Pereopod VII (Fig. 19F, K): coxal plate with four setae on posterior margin; basis elongated, with five setae and four spines on anterior margin, anterodistal corner with two spines; posterior margin with a row of ten setae; merus and carpus with one or two groups of spines on anterior margins, and two or one spine on posterior margins; propodus with three groups of spines on anterior margin and a seta on posterior margin; dactylus with one seta at hinge of unguis.

Coxal gills (Figs 18B, 19B, C, E, F): present on gnathopod II and pereopods III–VII, sac-like.

**Pleon.** Epimeral plates (Fig. 17B): plate I ventrally rounded, with six setae on anteroventral margin and two setae on posterior margin; plate II with two spines on ventral margin, posterior margin blunt, with one seta; plate III with two spines on ventral margin, posterior margin acute, with two setae.

Pleopods (Fig. 18F–H): subequal, peduncle with some long setae, bearing two retinacula accompanied by two setae; rami with approximately 18 segments, fringed with plumose setae.

**Urosome.** Urosomites (Fig. 17C): non-humped, urosomite I with two-one-one-two spines on dorsal margin; urosomite II with three-one-one-two spines on dorsal margin; urosomite III with two and one spine on lateral margins respectively, and two setae on dorsal margin.

Uropod I (Fig. 20D): peduncle with one basofacial spine, outer margin with one spine on outer margin, two spines on outer distal corner, one spine on inner margin, and one spine on inner distal corner; outer ramus with one spine on inner and outer margins each; inner ramus with one mid-lateral spine, both inner and outer rami with five distal spines.

Uropod II (Fig. 20E): peduncle with one spine on outer margin, and one spine on outer and inner corners each; outer ramus with one spine on outer margin; inner ramus with one spine on inner margin, both rami with five distal spines.

Uropod III (Fig. 20G): peduncle with one marginal spine and three distal spines; inner ramus 0.6 times the length of outer ramus, with two marginal and two distal spines; proximal article of outer ramus with three pairs of spines on outer margin, one spine on inner margin and four distal spines; terminal article of outer ramus longer than adjacent spines; both rami with few marginal setae.

Telson (Fig. 20F): cleft, each with three or four distal spines accompanied by few short setae.

##### Description of paratype female.

(IZCAS-I-A0063-2), 5.5 mm.

**Pereon.** Gnathopod I (Fig. 21A, C): palm of propodus not oblique as that of male, with eight spines on posterior corner.

Gnathopod II (Fig. 21B, D): carpus approx. as long as propodus, propodus subrectangular, palm truncated, with four spines on posterior corner.

Pereopods III–VII (Fig. 22A–J): similar to those of male. Pereopods III and IV with more setae on posterior margins; pereopods V–VII stouter, basis broader than those of male.

Oostegites (Figs 20A, B, 22B, C): progressively decreasing in size, with long marginal setae.

**Urosome.** Uropod III (Fig. 20C): stouter than that of male, inner ramus less than half the length of outer ramus; both rami with few setae.

##### Habitat.

This species was collected along a riverside with altitude 4400 m, water polluted.

##### Remarks.

*Gammarus limosus* sp. n. is similar to *G. altus* sp. n. in antenna II having calceoli; pereopod V having few setae on posterior margin; and bases of pereopods V–VII elongated. *Gammarus limosus* sp. n. can be distinguished from *G. altus* (*G. altus* in parentheses) by pereopod III with few setae on posterior margin (merus with four groups of setae on posterior margin); epimeral plate III acute on posterodistal corner (blunt); and uropod III inner ramus longer than half of outer ramus length (inner ramus 0.3 times the length of outer ramus).

*Gammarus limosus* sp. n. is very similar to *G. balcanicus* Schäferna, 1922 (widespread in Europe). It differs from the latter by pereopods III–IV and uropod III having few setae; bases of pereopods VI and VII slender.

The species of the genus *Gammarus* recorded from the Tibetan Plateau can be classified into four groups based on morphological comparison: (1) *G. lacustris* group, is characterized by uropod III inner ramus longer than half of outer ramus length, both rami fringed with plumose setae, and includes five species: *G. lacustris* Sars, 1863, *G. lasaensis* Barnard & Dai, 1988, *G. hongyuanensis* Barnard & Dai, 1988, *G. frigidus* Hou & Li, 2004, and *G. jaspidus* Hou & Li, 2004; (2) cave species with no eyes, including *G. abstrusus* Hou, Platvoet & Li, 2006, and *G. praecipuus* Li, Hou & An, 2013; (3) *G. sinuolatus* Hou & Li, 2004 with long simple setae on uropod III; and (4) *G. kangdingensis* group is characterized by uropod III having a few simple or plumose setae, and includes *G. emeiensis* Hou, Li & Koenemann, 2002, *G. sichuanensis* Hou, Li & Zheng, 2002, *G. glaber* Hou, 2017, *Gammarus altus* sp. n., *G. kangdingensis* sp. n., *G. gonggaensis* sp. n., and *G. limosus* sp. n. A key to these species is presented as follows.

### Key to the species of *Gammarus* from the Tibetan Plateau

**Table:** 

1	Eyes absent	**2**
–	Eyes presen	**3**
2	Pereopod III with long curled setae on posterior margin	***G. abstrusus***
–	Pereopod III with short setae on posterior margin	***G. praecipuus***
3	Uropod III inner ramus longer than half of outer ramus, both rami with dense plumose setae	**4**
–	Uropod III with some simple or plumose setae	**8**
4	Epimeral plates II and III very acute on posterodistal corners	**5**
–	Epimeral plate II and III moderate on posterodistal corners	**6**
5	Gnathopod II propodus palm with three medial spines	***G. lasaensis***
–	Gnathopod II propodus palm with one medial spine	***G. lacustris***
6	Urosomites dorsally elevated	***G. jaspidus***
–	Urosomites dorsally non-humped	**7**
7	Uropod III inner ramus reaching 0.8 times the length of outer ramus	***G. frigidus***
–	Uropod III inner ramus reaching 0.6 times the length of outer ramus	***G. hongyuanensis***
8	Uropod III only armed with long simple setae	***G. sinuolatus***
–	Uropod III with simple or plumose setae	**9**
9	Pereopods III and IV merus and carpus with long setae on posterior margins	**10**
–	Pereopods III and IV merus and carpus with a few short setae on posterior margins	**12**
10	Antenna II peduncle with long setae, calceoli absent	**11**
–	Antenna II peduncle with short setae, calceoli present	***G. kangdingensis* sp. n.**
11	Uropod III inner ramus reaching 0.4 times the length of outer ramus	***G. gonggaensis* sp. n.**
–	Uropod III inner ramus reaching 0.7 times the length of outer ramus	***G. emeiensis***
12	Uropod III inner ramus reaching 0.6 times the length of outer ramus, both rami with plumose setae	***G. sichuanensis***
–	Uropod III with few plumose setae	**13**
13	Pereopods V–VII bases elongated	**14**
–	Pereopods V–VII bases broad	***G. glaber***
14	Uropod III inner ramus longer than half of outer ramus	***G. limosus* sp. n.**
–	Uropod III inner ramus 0.3 times the length of outer ramus	***G. altus* sp. n.**

## Supplementary Material

XML Treatment for Gammarus

XML Treatment for Gammarus altus

XML Treatment for Gammarus kangdingensis

XML Treatment for Gammarus gonggaensis

XML Treatment for Gammarus limosus
